# Mitochondrial Fitness as a Functional Immune Checkpoint in Cancer: Metabolic Plasticity, Tumor Evolution, and Immunotherapy

**DOI:** 10.3390/cancers18172818

**Published:** 2026-09-01

**Authors:** Fortunato Morabito, Enrica Antonia Martino, Antonella Bruzzese, Nicola Amodio, Ernesto Vigna, Massimo Gentile

**Affiliations:** 1 Dafne Srl, 89048 Reggio Calabria, Italy; f.morabito53@gmail.com; 2 Hematology Unit, Department of Onco-Hematology, AO Hospital of Cosenza, 87100 Cosenza, Italy; enricaantoniamartino@gmail.com (E.A.M.); a.bruzzese@aocs.it (A.B.); ernesto.vigna@aocs.it (E.V.); 3 Department of Experimental and Clinical Medicine, University Magna Graecia of Catanzaro, 88100 Catanzaro, Italy; amodio@unicz.it; 4 Department of Pharmacy, Health and Nutritional Science, University of Calabria, 87036 Rende, Italy

**Keywords:** mitochondria, cancer metabolism, tumor microenvironment, immune escape, T-cell exhaustion, immunotherapy, CAR-T cells, bispecific antibodies, oxidative phosphorylation, precision oncology

## Abstract

Mitochondria are increasingly recognized as dynamic regulators of cancer-cell adaptation, immune function, and therapeutic response. Beyond their canonical role in energy production, mitochondrial metabolism, dynamics, quality control, and stress signaling influence tumor-cell survival and the capacity of immune effector cells to sustain antitumor activity within the tumor microenvironment. In this narrative review, we examine mitochondrial fitness as a multidimensional functional property encompassing bioenergetic capacity, metabolic flexibility, redox homeostasis, mitochondrial quality control, and adaptation to cellular and therapeutic stress. We propose the mitochondrial functional immune checkpoint as a conceptual framework linking mitochondrial fitness in malignant and immune cells to tumor–immune interactions and immunotherapy response. We discuss how mitochondrial metabolic plasticity, mitochondrial stress and mtDNA signaling, reactive oxygen species, mitochondrial dynamics, and intercellular mitochondrial transfer contribute to immune escape and treatment resistance. We further examine the relevance of mitochondrial fitness to immune checkpoint blockade, CAR-T-cell therapy, and T-cell-redirecting bispecific antibodies, with particular attention to hematological malignancies, including acute myeloid leukemia and multiple myeloma, while incorporating selected evidence from solid tumors to highlight shared mitochondrial mechanisms and their broader oncologic relevance. Finally, we discuss emerging strategies for mitochondrial targeting and functional mitochondrial profiling and their potential integration with established molecular and measurable residual disease assessments. Current evidence supports mitochondrial biology as a complementary dimension of precision oncology, although important challenges remain regarding context dependence, biomarker standardization, therapeutic selectivity, and preservation of immune-cell fitness. Prospective studies are needed to determine whether functional mitochondrial profiling can improve patient stratification and guide rational therapeutic combinations that selectively exploit tumor mitochondrial vulnerabilities while preserving effective antitumor immunity.

## Simple Summary

This review proposes the mitochondrial functional immune checkpoint as a conceptual framework linking mitochondrial fitness to cancer–immune interactions. Rather than acting through conventional ligand-receptor signaling, mitochondrial fitness represents an organelle-level regulatory layer influencing both tumor-cell adaptation and immune-cell competence. In cancer cells, metabolic plasticity and mitochondrial activity sustain oxidative phosphorylation, stress tolerance, stem-like properties, and resistance to treatments such as immune checkpoint inhibitors, CAR-T cell therapies, and bispecific antibodies. Conversely, in immune effectors, mitochondrial dysfunction contributes to T-cell exhaustion and reduced functional persistence within the hostile tumor microenvironment. Consequently, effective therapeutic strategies require a dual approach: selectively exploiting tumor-specific mitochondrial vulnerabilities while preserving or enhancing the bioenergetic competence of immune cells. Integrating functional mitochondrial profiling into precision oncology can optimize biomarker development, clarify resistance mechanisms, and improve the durability of immunotherapy responses.

## 1. Introduction

Cancer is increasingly understood as an evolutionary and ecological process in which malignant cells continuously adapt to selective pressures imposed by their microenvironment, the immune system, and anticancer therapy. Although genomic alterations remain central to oncogenesis and tumor classification, they do not fully account for the dynamic biological states that determine disease progression, metastatic competence, therapeutic resistance, or immune escape. Mitochondria are central to these adaptive processes because they integrate energy production with redox control, biosynthesis, apoptosis, inflammatory signaling, and epigenetic regulation [1,2].

The Warburg effect established that many cancer cells maintain high glycolytic flux despite oxygen availability [3]. However, this observation should not be interpreted as evidence that tumors universally lack functional mitochondria. Many cancers retain substantial mitochondrial activity and use oxidative phosphorylation, tricarboxylic-acid-cycle intermediates, and mitochondrial redox pathways to support proliferation, biomass generation, stress resistance, and metastatic dissemination [4,5]. Glycolysis and mitochondrial respiration are therefore not mutually exclusive programs; rather, their relative contribution varies across tumor types, microenvironmental niches, and phases of disease evolution.

Mitochondrial plasticity includes changes in organelle biogenesis, fusion and fission, mitophagy, mitochondrial DNA maintenance, substrate utilization, and respiratory capacity. These processes influence whether cancer cells proliferate, enter quiescence, survive metabolic stress, undergo apoptosis, or acquire therapy-resistant states [6,7,8].

Mitochondrial plasticity and mitochondrial fitness are related but distinct concepts. Mitochondrial plasticity describes the capacity of the mitochondrial network to remodel its structure and function in response to changing conditions, whereas mitochondrial fitness refers to the capacity of a cell to maintain or adapt mitochondrial bioenergetic, redox, biosynthetic, signaling, and stress-response functions under metabolic, immune, and therapeutic pressure. Mitochondrial fitness should therefore not be equated with high oxidative phosphorylation or maximal respiratory activity; rather, it represents a context-dependent functional phenotype reflecting the ability of mitochondria to preserve or reconfigure essential functions as cellular demands and selective pressures change [9].

In hematological malignancies, mitochondrial dependence is particularly relevant. Leukemia stem cells may rely on oxidative metabolism and amino-acid-supported mitochondrial respiration, creating vulnerabilities that can be therapeutically exploited [10,11]. Similarly, multiple myeloma cells depend on coordinated mitochondrial metabolism, proteostasis, and redox regulation to sustain their secretory phenotype and survive treatment-induced stress [12,13].

These observations nevertheless illustrate context-dependent mitochondrial vulnerabilities rather than a universal metabolic phenotype. The extent and nature of mitochondrial dependence can vary according to tumor lineage, oncogenic genotype, differentiation state, microenvironmental conditions, and previous treatment exposure.

At the same time, immunotherapy has transformed the treatment of several solid and hematological malignancies. Immune checkpoint inhibitors, CAR-T cells, and bispecific antibodies can restore or redirect antitumor immune responses and may produce deep, durable remissions in selected patients [14,15]. Nevertheless, primary resistance, incomplete responses, and relapse remain frequent. Established mechanisms include antigen loss, defective antigen presentation, immune exclusion, inhibitory signaling, clonal evolution, and an immunosuppressive tumor microenvironment. Metabolic constraints within the tumor microenvironment represent an additional layer of resistance, influencing whether immune effector cells can sustain the energetic, biosynthetic, and redox demands of prolonged antitumor activity.

Antitumor T cells must sustain proliferation, migration, cytokine secretion, and cytotoxic function in a microenvironment characterized by hypoxia, nutrient depletion, lactate accumulation, oxidative stress, and competition with malignant and stromal cells [16]. These conditions can impair mitochondrial biogenesis, respiratory capacity, and metabolic flexibility, thereby promoting dysfunctional or exhausted immune states. In tumor-infiltrating T cells, impaired mitochondrial fitness is associated with reduced antitumor activity and diminished capacity to respond to sustained antigenic stimulation [17,18].

Recent experimental evidence further indicates that mitochondrial metabolism actively shapes CD8^+^ T-cell differentiation and exhaustion. In particular, alterations in mitochondrial metabolism can promote dysfunctional T-cell states and reduce tumor sensitivity to immunotherapy, whereas metabolic interventions that preserve mitochondrial fitness can enhance T-cell persistence and antitumor activity [19,20].

Conversely, mitochondrial adaptation in tumor cells can contribute to immune escape. Mitochondria influence reactive oxygen species signaling, mitochondrial DNA release, innate immune activation, redox balance, cellular stress responses, and the production of metabolites that reshape immune-cell behavior [21,22]. Intercellular mitochondrial transfer represents an additional mechanism through which mitochondrial states may be exchanged within the tumor microenvironment, potentially altering the metabolic fitness of tumor, stromal, and immune cells. Recent evidence indicates that tumor-derived mitochondria can be transferred to tumor-infiltrating T cells, resulting in metabolic abnormalities, senescence, and impaired effector and memory functions [23]. Thus, mitochondrial biology affects both sides of the cancer-immune interaction: it can increase tumor-cell fitness under immune pressure while simultaneously constraining the functional competence of immune effector cells.

For this reason, we propose the mitochondrial functional immune checkpoint as a conceptual framework rather than as a new receptor–ligand signaling system analogous to PD-1/PD-L1 or CTLA-4. It describes a functional constraint shaped by the mitochondrial state of malignant and immune cells, influencing whether tumor cells can withstand metabolic and immune pressure and whether immune effector cells can sustain antitumor activity. The framework is therefore permissive or limiting rather than binary, reflecting the dynamic balance between mitochondrial competence and cellular stress within the tumor–immune ecosystem. This perspective may help explain why apparently favorable features, such as antigen expression or immune-cell infiltration, do not always translate into durable clinical benefit.

Recent reviews have surveyed mitochondrial metabolic reprogramming in cancer and immune cells and the emerging biology of intercellular mitochondrial transfer within the tumor microenvironment [24,25]. This review builds on this literature by focusing on mitochondrial fitness as an integrative functional phenotype linking tumor adaptation with immune-cell competence. Rather than considering mitochondrial metabolism solely as a determinant of tumor-cell bioenergetics, we examine how mitochondrial plasticity, quality control, redox regulation, metabolic competition, and intercellular mitochondrial communication shape the capacity of tumor and immune cells to withstand selective pressure, with particular emphasis on immune checkpoint inhibitors, CAR-T cells, bispecific antibodies, and functional mitochondrial biomarkers.

The Review deliberately adopts a hematology-centered translational perspective rather than attempting a comprehensive survey of mitochondrial biology across all cancer types. This focus reflects the particularly well-defined links among mitochondrial dependence, measurable residual disease, bone-marrow microenvironmental interactions, and T-cell-redirecting therapies in hematological malignancies. Nevertheless, the underlying biological framework is not intended to be disease restricted. Throughout the Review, selected evidence from solid tumors is integrated where it provides mechanistic or pharmacological support for shared principles of mitochondrial plasticity, tumor–immune metabolic crosstalk, and treatment adaptation.

**Figure 1:**
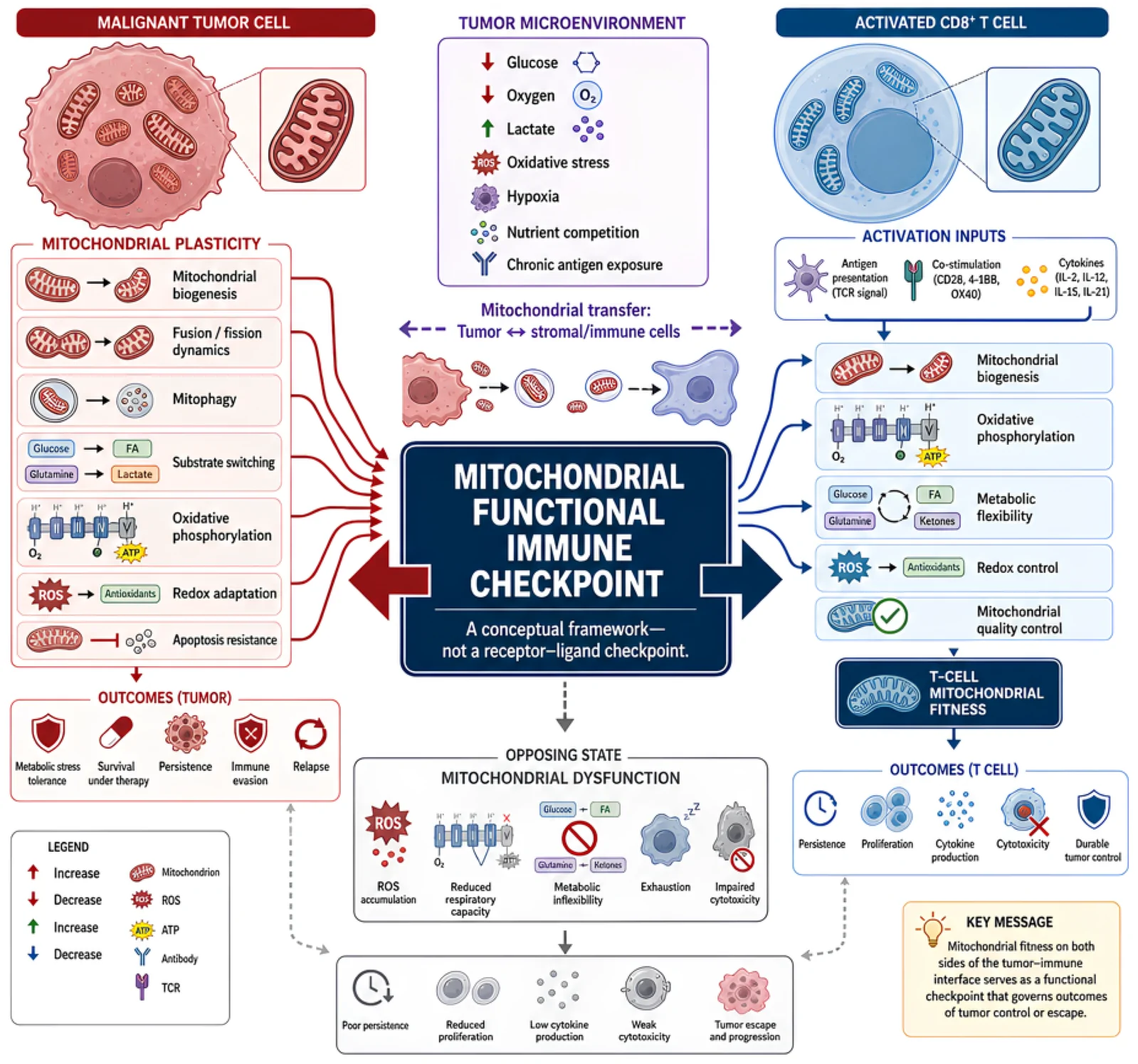
Mitochondrial plasticity at the interface of tumor adaptation and antitumor immunity. Mitochondria integrate metabolic, redox, stress, and apoptotic signals and dynamically adapt to nutrient availability, hypoxia, oxidative stress, and therapeutic pressure. In tumor cells, this plasticity can support survival and treatment resistance, whereas in immune cells mitochondrial competence influences activation, persistence, and effector function. These reciprocal effects contribute to the tumor–immune metabolic ecosystem and provide the basis for the mitochondrial functional immune-checkpoint concept.

The following sections examine mitochondrial plasticity as a driver of cancer evolution, mitochondrial dysfunction in immune escape and T-cell exhaustion, the relevance of mitochondrial fitness to CAR-T cells and bispecific antibodies, emerging therapeutic strategies, and the potential integration of mitochondrial biomarkers into functional precision immuno-oncology. The conceptual framework linking tumor mitochondrial adaptability with immune-cell mitochondrial competence is summarized in Figure 1. Hematological malignancies serve as the principal translational framework, while selected solid-tumor evidence is incorporated where it reinforces or extends the proposed concepts.

## 2. Mitochondrial Plasticity as a Driver of Cancer Evolution

Cancer cells do not possess a single, fixed mitochondrial phenotype. Instead, mitochondrial networks continuously remodel their structure, metabolism, quality-control pathways, and interactions with other organelles in response to nutrient availability, oxygen tension, oncogenic signaling, immune pressure, and anticancer treatment. This adaptive capacity—here referred to as mitochondrial plasticity—provides a mechanistic basis for context-dependent mitochondrial fitness. Importantly, plasticity should not be interpreted as intrinsically beneficial or equivalent to increased mitochondrial activity; rather, its biological consequence depends on whether remodeling restores cellular homeostasis, promotes survival, or facilitates adaptation to selective pressure [26,27,28].

### 2.1. Beyond the Warburg Effect: Mitochondrial Oxidative Metabolism in Cancer

Otto Warburg’s description of aerobic glycolysis established the principle that cancer cells can metabolize glucose through glycolysis despite adequate oxygen availability [3]. This observation was initially interpreted as evidence that mitochondrial respiration was defective in cancer. It is now clear, however, that many tumors retain functional mitochondria and can rely substantially on oxidative phosphorylation, particularly during nutrient stress, metastatic dissemination, therapeutic exposure, or transition to a slow-cycling state [29,30].

Glycolysis and oxidative phosphorylation should therefore not be viewed as mutually exclusive metabolic programs. Instead, cancer cells may use both pathways simultaneously or switch between them according to biological context. Glycolysis can support rapid biomass production and adaptation to hypoxic niches, whereas mitochondrial respiration may provide efficient ATP production, redox flexibility, and access to tricarboxylic-acid-cycle metabolites. This metabolic flexibility allows malignant cells to adapt their energy production to changing environmental conditions and contributes to functional heterogeneity within the tumor ecosystem.

In aggressive disease, increased oxidative metabolism is often observed in slow-cycling, invasive, metastatic, or treatment-resistant cell populations. Mitochondrial respiration may be particularly important when glycolytic substrates are limited or when cells require sustained energy production during migration, colonization, and stress adaptation [27,30]. Increased oxidative phosphorylation should not be considered synonymous with increased mitochondrial fitness. Rather, mitochondrial fitness reflects the capacity to dynamically adjust mitochondrial metabolism and maintain bioenergetic, redox, and stress-response functions as environmental and therapeutic pressures change. Thus, cancer cells may achieve a fitness advantage not simply by increasing oxidative phosphorylation, but by retaining the flexibility to redistribute metabolic flux between glycolysis, oxidative phosphorylation, fatty-acid oxidation, and amino-acid metabolism.

Hematological malignancies provide important examples of mitochondrial dependence. Acute myeloid leukemia stem cells can display increased reliance on oxidative phosphorylation and amino-acid-supported mitochondrial metabolism compared with more differentiated cell populations [10]. In multiple myeloma, the secretory burden of malignant plasma cells creates dependence on mitochondrial metabolism, proteostasis, and antioxidant pathways [12].

These observations illustrate context-dependent mitochondrial vulnerabilities rather than a universal metabolic phenotype. The contribution of mitochondrial respiration to cancer-cell fitness can vary according to lineage, differentiation state, oncogenic alterations, microenvironmental conditions, and treatment history.

Beyond oxidative metabolism itself, mitochondrial fitness is also influenced by the capacity of mitochondria to integrate metabolic and signaling functions. The mitochondrial outer membrane, for example, contains voltage-dependent anion channels (VDACs), which regulate the exchange of metabolites, nucleotides, and ions between mitochondria and the cytosol and thereby contribute to the coordination of glycolytic and mitochondrial metabolism [31,32]. Mitochondria also interact dynamically with the endoplasmic reticulum through mitochondria-associated membranes, which regulate calcium transfer, lipid exchange, and metabolic signaling. These processes can influence mitochondrial respiration, reactive oxygen species production, and susceptibility to mitochondrial-mediated cell death [6]. Such mechanisms further support a multidimensional definition of mitochondrial fitness that extends beyond measurements of oxidative phosphorylation alone.

### 2.2. Mitochondrial Dynamics and Quality Control, and Tumor-Cell Adaptation

Mitochondria are dynamic organelles that undergo fusion, fission, biogenesis, intracellular trafficking, and selective autophagic degradation through mitophagy. These coordinated processes regulate mitochondrial morphology, content, quality, and function, enabling cells to match their bioenergetic capacity to changing metabolic demands [6,33].

Mitochondrial dynamics and quality control should be considered integral components of mitochondrial fitness, because they determine the capacity of the mitochondrial network to maintain functional organelles while adapting to metabolic and cellular stress.

Mitochondrial fusion can support oxidative metabolism by forming interconnected mitochondrial networks, facilitating metabolite exchange, and preserving respiratory capacity during stress. In contrast, fission generates smaller mitochondrial units that can be distributed during cell division, transported to areas of high energy demand, or removed if damaged. In cancer, the biological effects of fusion and fission are context-dependent. Both processes can be co-opted to promote proliferation, migration, stress tolerance, metastatic behavior, and treatment resistance [6,34]. Thus, neither increased fusion nor increased fission should be interpreted per se as a marker of greater mitochondrial fitness. Rather, the ability to dynamically regulate the balance between these processes may allow cancer cells to preserve mitochondrial function while adapting to changing environmental conditions.

Mitophagy provides an additional layer of mitochondrial quality control. By eliminating damaged mitochondria, mitophagy can limit excessive reactive oxygen species production and preserve cellular homeostasis. However, its consequences in cancer are context-dependent [35]. Mitophagy may suppress malignant transformation in some settings by preventing oxidative damage and genomic instability, whereas in established tumors it may promote survival by preserving mitochondrial quality during hypoxia, nutrient deprivation, or treatment-induced stress [36]. Accordingly, mitochondrial fitness depends not simply on the abundance of mitochondria, but on the capacity to balance mitochondrial biogenesis, remodeling, and selective removal of dysfunctional organelles.

Mitochondrial architecture provides an additional level of regulation. Remodeling of the inner mitochondrial membrane and cristae can influence the organization of respiratory-chain complexes, ATP synthase activity, and the accessibility of pro-apoptotic proteins, thereby linking bioenergetic function to susceptibility to mitochondrial-mediated cell death [37]. Mitochondria also form dynamic contact sites with the endoplasmic reticulum and other organelles, coordinating calcium transfer, lipid exchange, and metabolic signaling. These contacts can influence mitochondrial respiration, reactive oxygen species production, and cellular stress responses [38]. Mitochondrial calcium handling is particularly relevant because controlled calcium uptake can stimulate oxidative metabolism, whereas excessive accumulation can promote permeability transition, loss of membrane potential, and cell death [6,38].

These processes are particularly relevant during anticancer therapy. Chemotherapy, targeted therapies [34,37,38,39,40,41,42,43,44], radiation, and immune-mediated cytotoxicity [8] can impose acute metabolic and oxidative stress, leading to mitochondrial dysfunction characterized by changes in mitochondrial morphology, membrane potential, respiratory activity, and reactive oxygen species production. Cancer cells that can rapidly remodel their mitochondrial network, remove damaged organelles, restore redox homeostasis, or redirect substrate utilization may therefore acquire a transient survival advantage during treatment.

Cells capable of restoring mitochondrial homeostasis, increasing antioxidant defenses, or shifting their substrate preference may survive as drug-tolerant persisters [45,46]. Such cells can later re-expand, acquire stable resistance mechanisms, and contribute to residual disease or clinical relapse [27,47]. This process provides a mechanistic link between mitochondrial plasticity and cancer evolution: therapeutic pressure may select cells with a greater capacity to restore mitochondrial function after injury rather than simply cells with constitutively high oxidative phosphorylation. In this context, mitochondrial fitness represents an adaptive phenotype that can facilitate survival during treatment and provide a substrate for subsequent clonal selection and disease recurrence.

### 2.3. Mitochondria, Cancer Stemness, and Disease Persistence

Cancer stem cells are operationally defined by their capacity for self-renewal, differentiation, tumor initiation, and disease regeneration after treatment. Although their metabolic phenotypes vary across cancer types, many stem-like populations demonstrate dependence on mitochondrial function. This does not mean that all cancer stem cells are uniformly OXPHOS-dependent; rather, they commonly exhibit metabolic flexibility and retain the ability to switch between glycolysis and mitochondrial respiration according to environmental conditions [48]. Rather than representing a uniformly OXPHOS-dependent population, cancer stem cells can dynamically balance glycolysis and mitochondrial metabolism according to nutrient availability, oxygen tension, differentiation state, and therapeutic pressure. This metabolic flexibility may itself represent an important component of their mitochondrial fitness and contribute to their capacity for long-term persistence.

Mitochondrial metabolism can promote stemness through several mechanisms. Oxidative phosphorylation supports ATP production in quiescent or nutrient-limited cells, whereas mitochondrial reactive oxygen species can influence signaling pathways linked to proliferation and differentiation. In addition, mitochondrial metabolites, including acetyl-coenzyme A, α-ketoglutarate, succinate, fumarate, and citrate, can modulate epigenetic enzymes and thereby influence chromatin accessibility and cell identity [49]. Mitochondria may therefore influence stemness not only by supplying energy, but also by regulating redox state, metabolite availability, epigenetic programs, and stress responses.

In leukemia, oxidative phosphorylation is particularly relevant to the persistence of stem-like populations. Leukemia stem cells can exhibit increased dependence on mitochondrial respiration and amino-acid metabolism compared with more differentiated leukemia cells [10].

This distinction is particularly relevant to venetoclax-based therapy. Venetoclax directly targets the anti-apoptotic protein BCL-2 and thereby lowers the apoptotic threshold of leukemia cells, while azacitidine induces broader changes in transcriptional and cellular-state programs. In AML, venetoclax plus azacitidine has been shown to disrupt energy metabolism and preferentially target leukemia stem cells, providing clinical evidence that metabolic state can influence therapeutic sensitivity [50]. Thus, the relationship between mitochondrial metabolism and venetoclax sensitivity is better understood as an interaction between metabolic state, mitochondrial function, and apoptotic dependence rather than as a direct consequence of hypomethylating therapy targeting mitochondrial respiration.

This distinction also highlights an important feature of mitochondrial fitness: therapeutic vulnerability may arise from the inability of a residual malignant population to remodel or restore mitochondrial function after treatment, rather than from constitutively elevated mitochondrial activity. Leukemia stem cells that preserve metabolic flexibility, maintain redox homeostasis, and efficiently remove damaged mitochondria may therefore have a selective advantage during treatment.

The clinical implication is important: conventional treatment may markedly reduce bulk tumor burden while sparing a metabolically adaptable residual population. Therefore, mitochondrial fitness may contribute not only to tumor growth but also to the biological capacity for disease recurrence.

In this setting, mitochondrial plasticity may help connect stemness with minimal residual disease and relapse by enabling residual leukemia cells to survive metabolic and therapeutic stress and subsequently regenerate the disease. However, direct evidence linking a prospectively measured mitochondrial-fitness phenotype to minimal residual disease and clinical relapse remains limited, and this represents an important area for future investigation.

Taken together, these observations support a model in which mitochondrial fitness contributes to cancer stemness primarily by providing metabolic and stress-adaptive capacity rather than by enforcing a fixed oxidative phenotype. This distinction is important for therapeutic development: strategies that disrupt mitochondrial adaptability or its interaction with apoptotic and epigenetic programs may preferentially target persistent stem-like populations while potentially limiting the emergence of treatment-resistant disease.

### 2.4. Mitochondrial Adaptation as a Mechanism of Therapeutic Resistance

Therapeutic resistance is one of the clearest examples of cancer evolution driven by mitochondrial plasticity. After exposure to anticancer therapy, surviving tumor cells may increase oxidative phosphorylation, alter mitochondrial mass, enhance antioxidant defenses, reprogram substrate utilization, or activate alternative anti-apoptotic pathways. Some of these changes are transient and reversible, whereas others become stable features of resistant clones [27]. Importantly, therapeutic resistance should not be interpreted simply as a consequence of increased mitochondrial activity. Rather, resistant cells may acquire the capacity to remodel mitochondrial metabolism, quality control, and apoptotic signaling in response to treatment-induced stress. This adaptive capacity represents a central component of mitochondrial fitness.

Mitochondrial adaptation may therefore involve several interconnected processes, including changes in respiratory activity, substrate utilization, redox buffering, mitochondrial turnover, and the threshold for mitochondrial apoptosis. Such adaptations can provide a temporary survival advantage, allowing drug-tolerant cells to persist during treatment and subsequently acquire or select for more stable resistance mechanisms.

Although hematological malignancies provide the principal disease models discussed below, mitochondrial adaptation to therapeutic pressure is not restricted to these diseases. Selected solid-tumor studies support the same broader principle that mitochondrial remodeling and metabolic plasticity can accompany treatment adaptation and persistence. The hematological examples should therefore be viewed as disease-specific manifestations of a broader cancer-adaptation framework rather than as evidence for a hematology-exclusive mechanism.

In selected settings, stromal mitochondrial donation may support recovery of tumor-cell bioenergetics after treatment-induced stress. In acute myeloid leukemia, the transfer of mitochondria from bone marrow stromal cells to leukemic cells has been associated with enhanced oxidative metabolism and protection during chemotherapy exposure [51].

This observation illustrates that mitochondrial fitness can also be supported by the tumor microenvironment rather than being determined exclusively by tumor-cell-intrinsic mechanisms. However, the extent to which mitochondrial transfer contributes to clinically relevant treatment resistance in patients remains incompletely established.

In acute myeloid leukemia, resistance to venetoclax plus azacitidine can arise through increased fatty-acid oxidation, which bypasses the dependence on amino-acid metabolism required to sustain oxidative phosphorylation in sensitive leukemia stem cells. Pharmacologic inhibition of fatty-acid oxidation restored sensitivity to venetoclax plus azacitidine in resistant experimental models [52]. This finding provides a particularly clear example of metabolic plasticity: when a therapeutically vulnerable metabolic state is disrupted, resistant cells can redirect substrate utilization to preserve mitochondrial function and evade apoptosis. Thus, fatty-acid oxidation may represent a compensatory mechanism that sustains mitochondrial fitness rather than simply a marker of increased oxidative phosphorylation.

In multiple myeloma, mitochondrial metabolic activity is closely integrated with endoplasmic-reticulum stress responses, proteasome function, oxidative stress control, and apoptotic regulation. These interactions may support resistance to proteasome inhibitors, immunomodulatory agents, and emerging immunotherapies [12].

The same principle may therefore extend across hematological malignancies, although the specific metabolic dependencies and adaptive mechanisms are likely to differ according to disease lineage, genetic background, treatment exposure, and microenvironmental context.

Recent evidence further extends this concept by demonstrating that metabolic pathways outside the electron transport chain can regulate mitochondrial fitness under therapeutic pressure. In AML, ADSS2, a key enzyme in de novo AMP biosynthesis, was shown to promote resistance to BH3 mimetics through regulation of AMP–AMPK signaling and mitophagy. Inhibition of ADSS2 impaired the mitochondrial quality-control response to BH3-mimetic-induced stress and restored sensitivity to venetoclax and MCL1 inhibition [53]. This finding is particularly relevant to the framework proposed here because it demonstrates that nucleotide metabolism can indirectly regulate mitochondrial quality control and apoptotic sensitivity.

The ADSS2 findings also broaden the concept of mitochondrial adaptation beyond oxidative phosphorylation. A cancer cell may maintain mitochondrial fitness not only by sustaining respiratory activity, but also by preserving the capacity to remove damaged mitochondria, buffer metabolic stress, and maintain an appropriate apoptotic threshold. Consequently, pathways that are not conventionally classified as “mitochondrial” may nevertheless determine whether mitochondria remain functionally competent during therapeutic stress [53].

**Figure 2:**
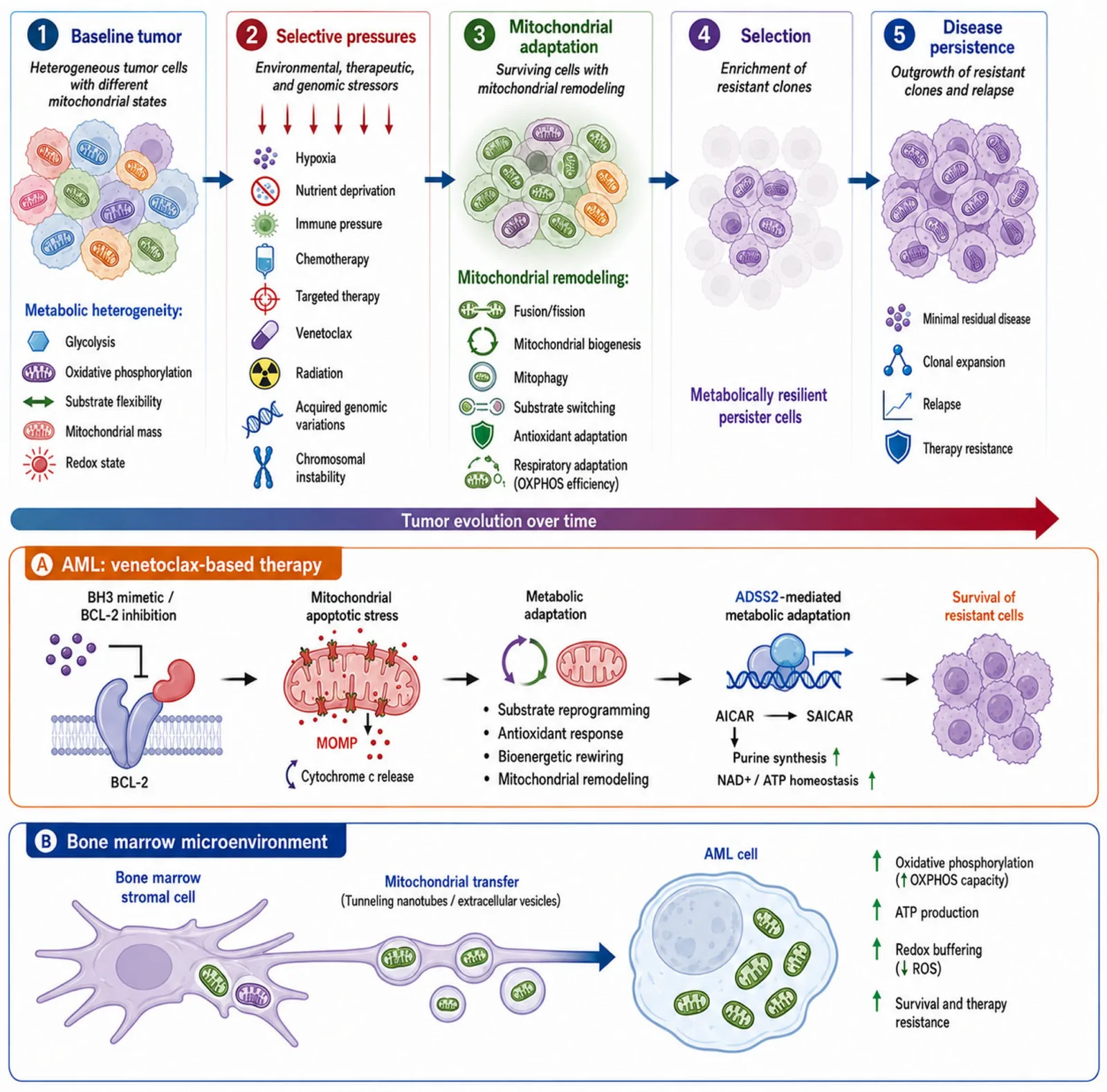
Mitochondrial plasticity as a driver of cancer adaptation and therapeutic resistance. Tumor cells dynamically remodel mitochondrial metabolism, dynamics, quality control, redox homeostasis, and substrate utilization in response to environmental and therapeutic stress. These adaptations can promote survival, persistence, and selection of metabolically resilient clones, contributing to treatment resistance and disease relapse.

Overall, mitochondrial plasticity should be viewed as an adaptive component of cancer evolution rather than simply as metabolic support for malignant growth. By dynamically regulating respiratory activity, substrate utilization, redox homeostasis, mitochondrial quality control, and apoptotic susceptibility, cancer cells can survive transient therapeutic stress and create a cellular reservoir from which resistant disease may emerge. The evolutionary relationship between environmental and therapeutic selective pressures, mitochondrial remodeling, metabolic resilience, and disease persistence is summarized in Figure 2.

Recent studies in hematologic malignancies further indicate that mitochondrial adaptation involves not only changes in metabolic activity but also remodeling of mitochondrial architecture and quality-control pathways. Alterations in mitochondrial fusion, fission, trafficking, mitochondrial transfer, and selective removal of damaged organelles can enable malignant cells to adapt mitochondrial function to environmental and therapeutic stress. These processes contribute to mitochondrial heterogeneity and can influence tumor–microenvironment interactions and treatment resistance [54]. By highlighting mitochondrial network remodeling as a dynamic component of cancer-cell adaptation, these findings identify mitochondrial architecture and quality-control pathways as potential therapeutic vulnerabilities that may be exploited to disrupt the programs sustaining malignant-cell survival and disease progression [55].

## 3. Mitochondria as Regulators of Immune Escape: The Functional Immune Checkpoint

Antitumor immunity depends on more than antigen recognition and receptor-mediated signaling. Effective immune responses require sufficient metabolic capacity to support T-cell activation, proliferation, migration, cytokine production, and cytotoxic killing within the tumor microenvironment. Conversely, cancer cells can remodel their metabolic environment to restrict nutrient availability, accumulate inhibitory metabolites, and generate oxidative and hypoxic stress. Mitochondria therefore influence cancer-immune interactions at multiple levels: they shape the metabolic ecosystem of the tumor, regulate cellular stress and inflammatory signaling, influence immune-cell fitness, and may contribute to mechanisms of immune escape.

The term mitochondrial functional checkpoint is used here as a conceptual framework rather than as a newly defined receptor-ligand pathway. Classical immune checkpoints, such as PD-1/PD-L1 and CTLA-4, regulate immune activation through defined inhibitory signaling interactions. By contrast, mitochondrial fitness determines whether both tumor cells and immune cells possess the metabolic capacity to survive, adapt, and sustain their biological functions. Thus, even when antitumor T cells recognize malignant cells and receive activating signals, their response may fail if their mitochondrial function is insufficient to support durable effector activity. Mitochondrial fitness should not be equated with a single parameter such as oxidative phosphorylation, mitochondrial mass, or membrane potential. Rather, it encompasses the capacity of mitochondria to maintain bioenergetic function, redox homeostasis, metabolic flexibility, and quality control under persistent cellular and therapeutic stress.

### 3.1. The Tumor Metabolic Ecosystem: Mitochondria at the Center of Immune Competition

The tumor microenvironment is a metabolically heterogeneous ecosystem in which malignant cells, stromal cells, endothelial cells, and immune populations compete for glucose, amino acids, oxygen, and other nutrients. Tumor cells can consume large quantities of glucose and thereby limit the availability of this substrate to infiltrating T cells. In experimental models, tumor-driven glucose restriction reduces T-cell glycolysis, mammalian target of rapamycin signaling, and interferon-γ production, thereby impairing antitumor immunity [16,54]. Mitochondrial metabolism contributes to this competition by influencing oxygen consumption, redox balance, and substrate utilization. In tumors with high respiratory activity, oxygen consumption may aggravate local hypoxia. Hypoxia can alter the metabolism and differentiation of both malignant and immune cells, promote the accumulation of immunosuppressive myeloid populations, and favor regulatory T-cell activity [56,57]. Lactate is another important mediator of metabolic immune suppression. It is not merely a glycolytic waste product: lactate accumulation and extracellular acidification can reduce T-cell proliferation, cytokine secretion, and cytotoxic activity while also supporting immunosuppressive myeloid-cell phenotypes [58]. Tumor mitochondrial flexibility can further reinforce this hostile environment by permitting cancer cells to switch among substrates and sustain growth despite variable nutrient availability. Thus, mitochondrial metabolism contributes to metabolic competition not only through ATP production but also through regulation of oxygen consumption, substrate utilization, redox balance, and adaptation to nutrient stress.

### 3.2. Mitochondrial Stress, Mitochondrial DNA, and Tumor Immunogenicity

Mitochondria link cellular stress to innate immune signaling. Under conditions of mitochondrial damage, altered mitochondrial DNA packaging or membrane integrity can enable mitochondrial DNA to reach the cytosol. Cytosolic mitochondrial DNA can activate cyclic GMP-AMP synthase and stimulator of interferon genes signaling, leading to interferon regulatory factor 3 activation and type I interferon responses [21]. In cancer, this signaling axis may have opposing consequences. Acute mitochondrial stress and mitochondrial DNA release may increase inflammatory signaling and potentially enhance immune recognition. In contrast, chronic mitochondrial dysfunction, defective mitochondrial quality control, or sustained inflammatory signaling may promote tumor adaptation, immune suppression, and treatment resistance. Therefore, the immunological consequences of mitochondrial stress depend on intensity, duration, cellular context, and the balance between immunostimulatory and immunosuppressive pathways. Mitochondria may also influence tumor visibility indirectly through their effects on reactive oxygen species, proteostasis, stress responses, and the availability of metabolites required for antigen processing and inflammatory pathways. However, the relationship between mitochondrial metabolism and antigen presentation is context-dependent and should not be presented as universally directional. Therefore, mitochondrial immunogenicity should be framed in this context as an emerging area that requires further mechanistic validation across diverse tumor types.

Mitochondrial damage signaling therefore represents an additional interface linking cellular metabolism, innate immunity, and immunotherapy response [59]. The consequences of this signaling are highly context-dependent: transient mtDNA release and cGAS–STING activation may enhance inflammatory signaling and tumor immunogenicity, whereas persistent mitochondrial stress may contribute to chronic inflammatory remodeling, immune dysfunction, and therapeutic resistance.

### 3.3. Reactive Oxygen Species as a Mitochondrial Rheostat of Immunity

Mitochondrial reactive oxygen species have dual roles in cancer and immunity. At physiological concentrations, they act as signaling molecules that support cellular activation, differentiation, and adaptation. At excessive concentrations, they can induce oxidative damage, disrupt protein and lipid homeostasis [60,61], impair mitochondrial respiration, and promote cell death or dysfunction [22,60]. Cancer cells frequently adapt to chronic oxidative stress by increasing antioxidant capacity and activating pathways that preserve redox homeostasis. These adaptations can improve tumor-cell survival during hypoxia, nutrient deprivation, chemotherapy, radiation exposure, and immune attack. However, tumor-associated oxidative stress can also impair T-cell receptor signaling, cytokine production, and cytotoxic function. Consequently, the balance between mitochondrial reactive oxygen species generation and antioxidant buffering can influence whether the tumor microenvironment supports immune activation or immune dysfunction. This balance is particularly relevant during immunotherapy. Checkpoint blockade can restore receptor-mediated activation in tumor-reactive T cells, but it may not correct profound mitochondrial dysfunction or oxidative stress. Conversely, restoration of mitochondrial fitness may improve the ability of T cells to sustain effector function following checkpoint inhibition.

Metabolic interventions should therefore be designed carefully. Broad suppression of reactive oxygen species may diminish immune-cell signaling, whereas uncontrolled oxidative stress may accelerate T-cell dysfunction or apoptosis. The therapeutic objective should therefore not be indiscriminate ROS suppression, but restoration of an appropriate redox range that preserves physiological immune signaling while limiting pathological oxidative stress.

### 3.4. Mitochondrial Dysfunction and T-Cell Exhaustion

T-cell exhaustion is a dysfunctional state that develops during chronic antigen exposure in cancer and persistent infection. Exhausted T cells progressively lose proliferative capacity, cytokine production, and cytotoxic function while acquiring distinct transcriptional, epigenetic, and metabolic characteristics [61,62]. Mitochondrial dysfunction is an important component of this process. Tumor-infiltrating T cells can display reduced mitochondrial biogenesis, abnormal mitochondrial morphology, impaired respiratory capacity, and limited metabolic flexibility [17,18]. Continuous antigen stimulation and hypoxia can jointly induce mitochondrial stress, repress peroxisome proliferator-activated receptor gamma coactivator-1α-dependent mitochondrial reprogramming, increase reactive oxygen species, and accelerate the acquisition of exhaustion-associated phenotypes [63]. These observations support the concept that mitochondrial dysfunction is not simply a consequence of T-cell exhaustion. It can contribute to the establishment and maintenance of exhaustion by limiting the capacity of T cells to respond to chronic antigen stimulation and metabolic stress. Mitochondrial dysfunction should not be considered synonymous with T-cell exhaustion. Exhaustion is a complex differentiation state shaped by chronic antigen exposure, inhibitory receptor signaling, transcriptional and epigenetic remodeling, and metabolic stress; mitochondrial dysfunction represents one component that can reinforce this phenotype.

Recent evidence further supports a direct role for mitochondrial integrity in sustaining antitumor T-cell function. Prothymosin alpha (PTMA) was shown to preserve mitochondrial DNA integrity and oxidative phosphorylation in CD8^+^ T cells under metabolic stress. PTMA was enriched in progenitor-exhausted T cells, whereas its loss impaired T-cell persistence and reduced the therapeutic efficacy of PD-1 blockade in experimental models [64].

Strategies that preserve mitochondrial quality, reduce excessive reactive oxygen species, improve oxygen availability, or support memory-associated metabolic programs may therefore enhance T-cell persistence and antitumor function.

### 3.5. Mitochondrial Transfer as an Emerging Modifier of Tumor–Immune Fitness

Intercellular mitochondrial transfer is an emerging mechanism through which tumor, stromal, and immune cells may modify one another’s metabolic state. Transfer can occur through tunneling nanotubes, extracellular vesicles, direct cell–cell contact, or uptake of extracellular mitochondrial material. In cancer, these processes may alter oxidative phosphorylation, redox balance, resistance to stress, and immune-cell function. However, their frequency, durability, and clinical relevance in human tumors remain incompletely defined. Preclinical studies indicate that cancer cells can acquire mitochondria from immune or stromal cells, improving metabolic fitness under stress. In a breast-cancer model, intercellular nanotubes mediated mitochondrial trafficking from immune cells to cancer cells, enhancing tumor-cell metabolic capacity while reducing immune-cell fitness; inhibition of this process improved the activity of PD-1 blockade [65]. Conversely, tumor-derived mitochondrial material may impair immune-cell competence. In melanoma and non-small-cell lung cancer, tumor-to-T-cell mitochondrial transfer has been associated with altered mitochondrial DNA status, mitochondrial dysfunction, and reduced antitumor immunity [66].

Recent evidence has strengthened the biological relevance of this phenomenon. A 2025 study demonstrated that mitochondria carrying tumor-associated mitochondrial DNA alterations can be transferred from cancer cells to tumor-infiltrating lymphocytes. These transferred mitochondria escaped normal mitophagic clearance, resulting in metabolic abnormalities, senescence, impaired effector function, and defective memory formation in recipient T cells. In clinical samples, the presence of tumor-associated mtDNA mutations was associated with poorer outcomes following immune checkpoint blockade in melanoma and non-small-cell lung cancer [23].

Conversely, recent experimental evidence indicates that mitochondrial transfer can also occur in the opposite direction, from immune cells to tumor cells. Tumor cells were shown to hijack mitochondria from immune cells, with mitochondrial loss impairing antigen presentation and the activation and cytotoxic capacity of NK and CD8^+^ T cells. In recipient tumor cells, transferred mitochondria fused with endogenous mitochondrial networks, promoted mtDNA leakage and cGAS–STING activation, and supported type I interferon-dependent immune-evasion programs. Interference with mitochondrial transfer or downstream cGAS–STING/type I interferon signaling reduced lymph node metastasis in experimental models [67,68].

Together with the stromal-to-leukemia mitochondrial transfer described in hematological malignancies, these solid-tumor observations indicate that intercellular mitochondrial trafficking is a cross-tumor phenomenon, although its direction, biological consequences, and therapeutic relevance differ according to the donor and recipient cell populations and the tumor context.

These findings suggest that mitochondrial transfer may contribute to immune escape by modifying the organelle fitness required for sustained T-cell activity. At present, this mechanism should be regarded as an emerging contributor to tumor–immune communication rather than a universally established determinant of immunotherapy resistance. Accordingly, mitochondrial transfer is relevant to the mitochondrial functional checkpoint because it may modify tumor or immune-cell fitness beyond cell-intrinsic metabolic reprogramming. Future studies should establish which donor–recipient pairs, transfer routes, and treatment contexts produce durable functional consequences and whether selective modulation of mitochondrial transfer can improve immunotherapy without impairing beneficial immune or stromal interactions.

### 3.6. The Mitochondrial Functional Immune-Checkpoint Concept

Taken together, these observations support the concept of a mitochondrial functional immune checkpoint. This checkpoint does not operate through a single ligand, receptor, or signaling axis. Instead, it represents the integrated mitochondrial capacity of malignant and immune cells to respond to metabolic stress, oxidative injury, chronic antigen stimulation, and therapeutic pressure. In tumor cells, mitochondrial fitness may influence the ability to maintain redox balance, survive nutrient restriction, resist apoptosis, and adapt to immune-mediated stress. In immune cells, mitochondrial fitness may influence whether immune activation can be sustained sufficiently to achieve durable tumor control. Therefore, effective immunotherapy may require both reduction in tumor-cell metabolic resilience and preservation or restoration of immune-cell metabolic competence. This framework may help explain why tumors with apparent antigenicity or immune-cell infiltration do not always respond durably to checkpoint inhibitors, CAR-T cells, or bispecific antibodies. Immune recognition is necessary but may be insufficient when tumor cells are metabolically adaptable or when effector T cells are already metabolically exhausted. Thus, mitochondrial biology extends beyond its conventional metabolic functions to represent an important regulator of tumor–immune interactions and immune surveillance.

**Figure 3:**
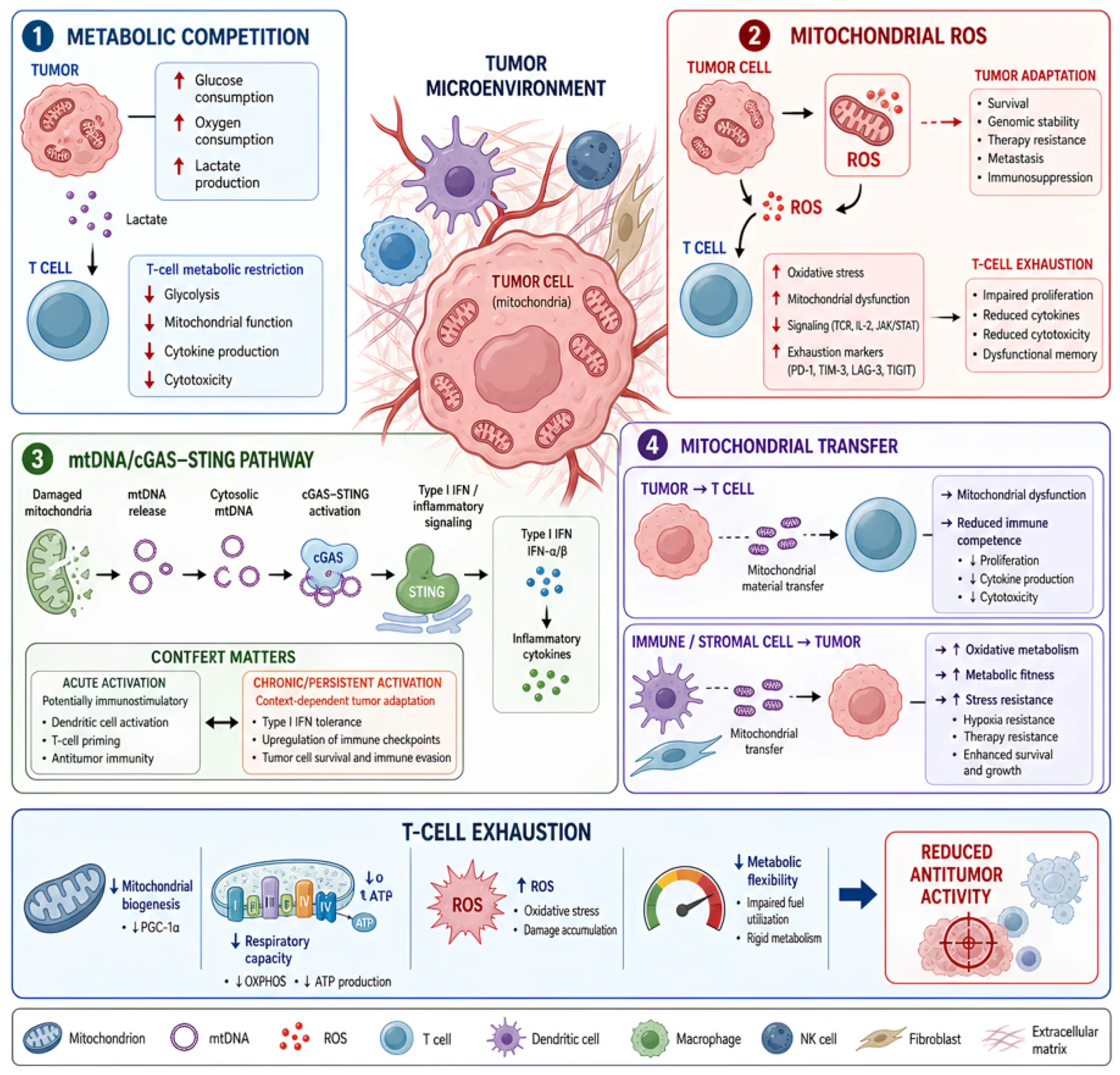
Mitochondrial fitness and immune-cell competence during immunotherapy. Antitumor T-cell activity requires metabolic flexibility, mitochondrial reserve, redox control, and adaptation to chronic antigenic and metabolic stress. Mitochondrial dysfunction can contribute to impaired persistence and exhaustion, whereas preservation or restoration of mitochondrial competence may support durable responses to immune checkpoint blockade, CAR-T-cell therapy, and T-cell-redirecting bispecific antibodies.

The major mechanisms linking mitochondrial fitness to tumor–immune interactions—including metabolic competition, mitochondrial ROS, mitochondrial DNA–dependent innate immune signaling, T-cell mitochondrial dysfunction, and intercellular mitochondrial transfer—are summarized in Figure 3 below.

Incorporating mitochondrial fitness into studies of immunotherapy resistance, biomarker development, and rational therapeutic combinations may therefore provide a more comprehensive framework for understanding why some tumors remain susceptible to immune elimination whereas others ultimately escape immune control. Importantly, the mitochondrial functional immune checkpoint is proposed here as a conceptual framework rather than as a clinically validated checkpoint or biomarker. Its translational relevance will require the identification of measurable features of mitochondrial fitness and validation of their predictive or therapeutic value across different immunotherapy settings.

## 4. Mitochondrial Fitness as a Determinant of Immunotherapy Success

T-cell activation is energetically demanding. Following antigen recognition, T cells must increase nutrient uptake, synthesize proteins and lipids, expand clonally, produce cytokines, migrate into tumor tissue, and execute cytotoxic functions. These processes require coordinated metabolic remodeling, including changes in glycolysis, oxidative phosphorylation, mitochondrial mass, mitochondrial dynamics, and redox regulation [23,69]. Thus, immune-cell activation alone is not sufficient to guarantee durable tumor control. Therapeutic benefit depends on whether activated immune cells retain the mitochondrial capacity required to persist, adapt to the tumor microenvironment, and maintain cytotoxic activity under chronic antigenic and metabolic stress. This principle may be particularly relevant to therapies that depend on prolonged T-cell engagement, including checkpoint blockade, CAR-T cells, and bispecific antibodies, although the strength of clinical evidence differs substantially among these platforms.

### 4.1. Mitochondrial Metabolism Defines T-Cell Fate and Antitumor Function

T-cell differentiation is closely linked to metabolic state. Naïve T cells predominantly use oxidative phosphorylation and fatty-acid oxidation to maintain quiescence and long-term survival. After activation, effector T cells increase glycolysis to support rapid proliferation and cytokine production. By contrast, memory T cells develop increased mitochondrial mass, spare respiratory capacity, and oxidative metabolic flexibility, enabling long-term survival and rapid responses after antigen re-exposure [67]. Mitochondrial remodeling is not merely a consequence of differentiation; it actively contributes to T-cell fate. Mitochondrial fusion, fatty-acid oxidation, and oxidative metabolism support the formation of memory-associated T-cell populations, whereas mitochondrial fission and glycolytic metabolism are associated with effector differentiation [70]. These states should not, however, be interpreted as rigid metabolic categories. Effective T-cell responses require dynamic transitions between glycolytic and oxidative programs, and mitochondrial fitness is better understood as the capacity to adapt these programs to changing functional demands.

Within tumors, chronic antigen exposure, hypoxia, nutrient competition, and oxidative stress can progressively impair mitochondrial function. Exhausted T cells may exhibit reduced mitochondrial biogenesis, decreased respiratory capacity, altered cristae organization, increased oxidative stress, and defective metabolic flexibility. These abnormalities limit their ability to sustain cytotoxic activity in the tumor microenvironment [17,63]. The latter study directly links continuous stimulation and hypoxia to mitochondrial stress and accelerated T-cell exhaustion. Immune checkpoint blockade should therefore not be interpreted as a universal reversal of exhaustion. PD-1 blockade can reinvigorate a subset of tumor-reactive T cells, particularly progenitor-like exhausted populations that retain proliferative potential, whereas terminally exhausted cells are less likely to recover durable functional capacity [71]. Mitochondrial competence may therefore help distinguish reversible immune dysfunction from more fixed states of terminal exhaustion. This distinction is relevant to the mitochondrial functional checkpoint proposed in this review: immune activation may restore signaling competence without necessarily restoring the mitochondrial capacity required for sustained effector function.

### 4.2. Mitochondrial Rejuvenation as a Strategy to Enhance Antitumor Immunity

The recognition that mitochondrial dysfunction contributes to immune failure has generated interest in approaches designed to preserve or restore immune-cell metabolic fitness. Potential strategies include enhancement of mitochondrial biogenesis, optimization of nutrient availability during ex vivo expansion, modulation of oxidative stress, preservation of mitochondrial quality control, and induction of memory-associated metabolic programs. Experimental studies indicate that interventions favoring oxidative metabolism and memory differentiation can improve T-cell persistence and antitumor function. For example, inhibition of glycolytic metabolism during T-cell activation can promote memory-cell formation and enhance antitumor activity in preclinical models [72]. These findings should not be interpreted as support for indiscriminate enhancement of mitochondrial activity in all immune settings.

Immune cells require dynamic metabolic adaptation, and the optimal intervention may differ according to disease type, immune cell subset, treatment phase, and microenvironmental context. The therapeutic objective should be to preserve metabolic flexibility and prevent irreversible mitochondrial dysfunction rather than simply increase oxidative phosphorylation.

This distinction is particularly important because mitochondrial activity can have context-dependent effects on immune function. Strategies that improve mitochondrial biogenesis or respiratory capacity may enhance persistence in some settings, whereas excessive oxidative stress or inappropriate metabolic reprogramming may impair effector function. Thus, “mitochondrial rejuvenation” should be understood as the restoration of functional reserve and metabolic flexibility rather than simply increasing mitochondrial activity.

### 4.3. CAR-T Cells: Mitochondrial Fitness as a Determinant of Persistence and Efficacy

CAR-T-cell therapy provides a unique opportunity to study and manipulate immune-cell metabolism because the therapeutic product is manufactured outside the patient before infusion. The phenotype, differentiation state, mitochondrial capacity, cytokine exposure, and metabolic characteristics of the infused product can all influence in vivo expansion, persistence, toxicity, and antitumor efficacy. CAR-T-cell products enriched for less differentiated, memory-associated T-cell states generally show superior proliferative capacity and persistence compared with highly differentiated effector populations. In chronic lymphocytic leukemia, sustained remission after CD19-directed CAR-T-cell therapy was associated with a CAR-T-cell product exhibiting features of memory differentiation and enhanced metabolic fitness [73]. The intracellular signaling architecture of the CAR also influences T-cell metabolism. CARs containing 4-1BB costimulatory domains promote central-memory-like characteristics, increased mitochondrial biogenesis, enhanced respiratory capacity, and fatty-acid oxidation. By contrast, CD28-based CARs favor stronger glycolytic activity and effector-memory differentiation [74]. These findings do not imply that one costimulatory domain is universally superior. CD28- and 4-1BB-based platforms can have distinct kinetics, expansion profiles, persistence patterns, and clinical roles. Nevertheless, they demonstrate that CAR design can directly shape the metabolic and mitochondrial state of therapeutic T cells. Future CAR-T-cell development may therefore benefit from deliberate metabolic engineering. Potential approaches include selection of T-cell subsets with memory-associated features, optimization of cytokine and nutrient conditions during manufacturing, control of tonic signaling, modulation of oxidative stress, and genetic interventions that preserve mitochondrial quality and metabolic flexibility. Mitochondrial features may also provide measurable characteristics for selecting or optimizing cellular products, although the clinical predictive value of individual mitochondrial parameters remains to be established.

Such approaches may be especially relevant in solid tumors and heavily pretreated hematological malignancies, where chronic antigen exposure and hostile metabolic conditions frequently impair CAR-T-cell persistence. Solid tumors provide a particularly stringent test of the mitochondrial-fitness concept because transferred T cells must maintain metabolic competence despite hypoxia, nutrient competition, stromal barriers, and sustained antigenic stimulation. Thus, although much of the clinically mature CAR-T experience discussed in this Review derives from hematological malignancies, the underlying mitochondrial principles may be especially relevant in solid-tumor settings.

The relationship between mitochondrial fitness and CAR-T efficacy is unlikely to be determined solely by the metabolic phenotype of the infused product. Following infusion, CAR-T cells encounter changing antigenic, inflammatory, and metabolic conditions that can progressively remodel mitochondrial function. Thus, strategies combining ex vivo optimization with preservation of mitochondrial fitness after infusion may ultimately be more effective than approaches focused exclusively on product manufacturing.

### 4.4. Bispecific Antibodies and Mitochondrial Constraints on Sustained Immune Activation

Bispecific antibodies redirect endogenous T cells toward tumor-associated antigens without requiring ex vivo cell manufacturing. In multiple myeloma, BCMA-directed and GPRC5D-directed bispecific antibodies have established T-cell redirection as an important therapeutic strategy. However, durable benefit is not universal, and disease progression can occur through tumor-intrinsic resistance, antigen modulation, impaired T-cell function, and progressive immune dysfunction [75,76]. Unlike CAR-T-cell therapy, bispecific antibodies depend on the patient’s endogenous immune repertoire. Their activity may therefore be influenced by baseline T-cell number, differentiation state, prior treatment exposure, immune aging, chronic inflammation, antigen burden, and the pre-existing metabolic fitness of circulating and tumor-resident T cells. Continuous T-cell engagement may also increase the risk of activation-associated dysfunction and exhaustion, particularly in settings of persistent antigen exposure. At present, however, direct clinical evidence linking mitochondrial dysfunction to resistance to bispecific antibodies remains limited. The mitochondrial contribution should therefore be considered a biologically plausible and testable mechanism rather than an established cause of treatment failure. This distinction is particularly important because resistance to bispecific antibodies is multifactorial and may reflect tumor-intrinsic mechanisms, antigen density or loss, T-cell exhaustion, immune-cell composition, and changes in the tumor microenvironment [77].

Prospective studies should evaluate whether mitochondrial respiratory capacity, mitochondrial stress signatures, oxidative damage, or exhaustion-associated transcriptional programs predict response duration or loss of response during bispecific-antibody therapy. Longitudinal sampling will be particularly informative because mitochondrial fitness may change during repeated T-cell engagement and disease evolution.

This distinction is important: mitochondrial profiling may ultimately identify patients whose endogenous T cells are capable of sustained redirection, but this hypothesis requires clinical validation before it can be incorporated into treatment-selection algorithms.

### 4.5. Clinical Implications

Mitochondrial fitness should be considered a potential determinant of immunotherapy outcome across treatment platforms. In checkpoint blockade, it may influence the capacity of tumor-reactive T cells to undergo productive reinvigoration. In CAR-T-cell therapy, it may influence the quality, persistence, and functional resilience of the manufactured cellular product. In bispecific-antibody therapy, it may influence whether endogenous T cells can tolerate repeated stimulation and maintain cytotoxic function. The translational objective is not to enhance mitochondrial metabolism indiscriminately. Instead, it is to identify tumor-specific mitochondrial vulnerabilities while maintaining, restoring, or engineering immune-cell mitochondrial competence. This dual strategy may help widen the therapeutic window of mitochondrial interventions and improve the depth and durability of immunotherapy responses. Achieving this goal will require approaches that distinguish tumor-cell mitochondrial dependencies from those required for immune-cell function and that account for the dynamic nature of mitochondrial fitness during treatment.

At present, mitochondrial fitness is best regarded as a candidate functional determinant and biomarker domain rather than a validated clinical selection tool. Future studies should therefore prioritize standardized measurements of mitochondrial function, longitudinal assessment during immunotherapy, and prospective validation of mitochondrial signatures against response, toxicity, and durability outcomes. Such studies could establish whether mitochondrial profiling can move beyond mechanistic description toward functional precision immuno-oncology.

## 5. Targeting the Mitochondrial Functional Checkpoint: Therapeutic Opportunities and Challenges

The recognition that mitochondrial fitness influences both tumor-cell survival and immune-cell competence raises a central therapeutic question: can mitochondrial vulnerabilities be exploited to selectively weaken cancer cells while preserving, or potentially enhancing, antitumor immunity? This question is challenging because mitochondria are essential to normal tissue homeostasis and immune-cell function. Broad inhibition of mitochondrial respiration may damage healthy tissues and impair the T cells required for successful immunotherapy. The therapeutic goal should therefore not be nonspecific mitochondrial suppression. Instead, treatment strategies should identify tumor-selective dependencies, exploit context-specific metabolic vulnerabilities, and avoid compromising the mitochondrial fitness of immune effector cells [30]. Cancer cells may exhibit mitochondrial liabilities that differ from those of normal tissues, including dependence on oxidative phosphorylation, mitochondrial translation, anti-apoptotic BCL-2-family proteins, altered redox control, and increased reliance on specific nutrient substrates. However, these vulnerabilities are heterogeneous across tumor types and may evolve during treatment. Mitochondrial targeting should therefore be guided by disease biology, treatment history, and, when possible, functional metabolic profiling.

Functional mitochondrial profiling remains an emerging approach rather than a clinically validated method for treatment selection. Its potential value lies in identifying tumor-specific dependencies and distinguishing them from mitochondrial programs required for immune-cell persistence and function.

### 5.1. Direct Targeting of Tumor Mitochondrial Metabolism

Many cancer cells use oxidative phosphorylation to support ATP synthesis, redox balance, anabolic metabolism, and survival under therapeutic stress. This dependence can be particularly pronounced in therapy-resistant, slow-cycling, stem-like, and metastatic tumor-cell populations. Pharmacologic inhibition of mitochondrial respiration has therefore emerged as a rational therapeutic approach. However, mitochondrial dependence is heterogeneous and should not be assumed to be a universal feature of cancer cells.

The complex I inhibitor IACS-010759 demonstrated that oxidative phosphorylation inhibition can exploit a metabolic vulnerability in cancer cells. In preclinical models, this agent reduced mitochondrial respiration and showed activity in tumors with increased oxidative metabolic dependence [78]. Clinical development has nevertheless highlighted the narrow therapeutic window of systemic OXPHOS inhibition, underscoring the distinction between demonstrating a metabolic vulnerability experimentally and translating that vulnerability into an effective therapy [79].

Another experimental example is Gboxin, a small molecule that targets mitochondrial oxidative phosphorylation through inhibition of the F0_0_F_1_ ATP synthase. Gboxin selectively impaired oxygen consumption and inhibited the growth of primary mouse and human glioblastoma cells, while a metabolically stable analogue reduced tumor growth in glioblastoma allograft and patient-derived xenograft models. These findings illustrate the potential of exploiting tumor-specific mitochondrial bioenergetic dependencies, but Gboxin remains a preclinical strategy and has not been established as a clinical anticancer therapy [80].

The glioblastoma findings also illustrate why selected solid-tumor models are informative within the present hematology-centered framework. Although specific mitochondrial dependencies and therapeutic windows are tumor-dependent, these studies provide complementary evidence that mitochondrial bioenergetic vulnerabilities can be therapeutically exploitable across disease boundaries. They should therefore be interpreted as support for the broader biological validity of mitochondrial targeting rather than as evidence that identical metabolic vulnerabilities or treatment strategies can be directly extrapolated between tumor types.

Acute myeloid leukemia provides a particularly relevant example. Leukemia stem cells can depend on mitochondrial oxidative phosphorylation and may be more sensitive to interventions that disrupt mitochondrial metabolism than more differentiated hematopoietic cells [9]. Nevertheless, OXPHOS inhibition remains challenging in the clinic. Tumors may compensate through increased glycolysis, alternative substrate use, mitochondrial biogenesis, or metabolic support from stromal cells. In addition, normal tissues with high oxidative requirements may limit the achievable therapeutic window. Consequently, OXPHOS inhibitors may be most effective when combined with agents that block compensatory pathways or when used in biologically selected patient populations.

From the perspective of the mitochondrial functional checkpoint proposed here, the critical issue is therefore not simply whether a tumor depends on oxidative phosphorylation, but whether its mitochondrial dependency can be selectively disrupted without compromising the mitochondrial function of antitumor immune cells. This consideration becomes particularly important when OXPHOS inhibitors are combined with immune checkpoint blockade, CAR-T cells, or bispecific antibodies.

### 5.2. Mitochondrial Translation and Protein-Synthesis Inhibitors

Mitochondrial oxidative phosphorylation requires the coordinated expression of nuclear and mitochondrial genes. Because mitochondrial DNA encodes essential components of the respiratory chain, cancer cells with high oxidative metabolic demand may be vulnerable to inhibition of mitochondrial protein synthesis. Mitochondrial ribosomes share evolutionary features with bacterial ribosomes, and some antibiotics can inhibit mitochondrial translation. In acute myeloid leukemia models, inhibition of mitochondrial translation sensitized venetoclax-resistant cells to venetoclax through mitochondrial respiratory dysfunction and activation of the integrated stress response. A genome-wide CRISPR screen identified mitochondrial translation as a vulnerability in venetoclax-resistant AML cells, while pharmacologic inhibition of mitochondrial protein synthesis with tedizolid or doxycycline restored venetoclax sensitivity and enhanced antileukemic activity in preclinical models [81]. This approach remains primarily preclinical and requires careful evaluation of toxicity, pharmacokinetics, duration of treatment, and the risk of damaging normal hematopoietic and immune cells. Because mitochondrial translation is also required for oxidative metabolism in normal tissues and immune cells, therapeutic selectivity will be critical, particularly when mitochondrial translation inhibitors are combined with immunotherapy. However, it illustrates an important principle: mitochondrial vulnerabilities can arise from the specific molecular machinery required to maintain respiration, rather than from respiratory-chain inhibition alone.

Mitochondrial quality control provides a complementary therapeutic strategy in which mitochondrial function is enhanced rather than suppressed. Urolithin A, a microbiome-derived metabolite that promotes mitophagy, has been shown to expand CD8^+^ T memory stem cells and enhance antitumor immunity in preclinical models. Urolithin A-induced mitophagy increased mitochondrial fitness in T cells and improved their antitumor activity, illustrating how modulation of mitochondrial quality control can potentially strengthen immune-cell competence rather than directly targeting tumor-cell respiration [82].

Urolithin A is also being investigated clinically, although its anticancer efficacy remains unproven. Its inclusion therefore illustrates an important distinction within the mitochondrial functional checkpoint framework: mitochondrial interventions may either suppress tumor-cell metabolic dependencies or enhance immune-cell mitochondrial competence, depending on the cellular target and biological context.

Mitochondrial proteostasis represents another emerging therapeutic target. ONC206, a more potent analogue of ONC201, activates the mitochondrial protease caseinolytic protease P (ClpP), resulting in excessive mitochondrial proteolysis, mitochondrial dysfunction, and cellular stress. Preclinical studies have demonstrated loss of mitochondrial membrane potential, impaired oxidative phosphorylation, increased mitochondrial reactive oxygen species, and induction of apoptosis following ONC206 exposure. ONC206 has subsequently entered early-phase clinical investigation in primary and recurrent central nervous system malignancies, although its clinical efficacy remains investigational [83].

In the context of the mitochondrial functional checkpoint proposed here, mitochondrial translation, mitophagy, and mitochondrial proteostasis represent mechanistically distinct therapeutic opportunities rather than interchangeable forms of “mitochondrial modulation.” Mitochondrial translation inhibitors and OXPHOS inhibitors aim to suppress tumor bioenergetic capacity, whereas urolithin A seeks to enhance mitochondrial quality control in immune cells and ONC206 exploits mitochondrial proteostasis in malignant cells. Whether these approaches can achieve sufficient cellular selectivity while preserving immune-cell mitochondrial competence remains an important unanswered question.

### 5.3. Targeting Mitochondrial Apoptosis: The BCL-2 Paradigm

The intrinsic apoptotic pathway is one of the most clinically successful examples of mitochondrial targeting. Mitochondrial outer-membrane permeabilization represents a critical point of commitment to apoptosis. This process is regulated by pro-apoptotic and anti-apoptotic members of the BCL-2 family, including BCL-2, BCL-XL, and MCL-1 [80]. Venetoclax exploits cancer-cell dependence on BCL-2 and has transformed the treatment of chronic lymphocytic leukemia and acute myeloid leukemia. In previously untreated older or medically unfit patients with acute myeloid leukemia, venetoclax combined with azacitidine improved remission rates and survival compared with azacitidine alone [84]. The clinical activity of venetoclax illustrates an important connection between mitochondrial metabolism and apoptosis: BCL-2 inhibition does not directly target oxidative phosphorylation, but alters the apoptotic threshold of cells whose survival may be closely linked to mitochondrial metabolic state.

In leukemia stem cells, venetoclax-based therapy can disrupt oxidative phosphorylation and energy metabolism. Studies in primary AML samples have shown that venetoclax plus azacitidine alters TCA-cycle metabolism and suppresses OXPHOS, providing a mechanistic link between BCL-2 dependence and mitochondrial bioenergetics [50]. Thus, mitochondrial respiration and BCL-2 dependence are functionally interconnected rather than independent vulnerabilities: metabolic state can influence apoptotic priming, while BCL-2 inhibition can perturb mitochondrial metabolism in susceptible leukemia cells. Resistance can nevertheless emerge through metabolic adaptation, increased fatty-acid oxidation, altered mitochondrial substrate use, and a shift in dependency from BCL-2 toward alternative anti-apoptotic proteins such as MCL-1 [52]. These findings reinforce the value of combining apoptosis-directed therapy with strategies that address the metabolic flexibility of resistant cells.

This relationship is particularly relevant to the mitochondrial functional checkpoint proposed in this review. A metabolically adaptable tumor cell may preserve mitochondrial function and avoid apoptosis despite BCL-2 inhibition, whereas disruption of complementary metabolic pathways may lower the apoptotic threshold and restore drug sensitivity. Conversely, therapeutic strategies targeting mitochondrial metabolism must account for the possibility of impairing mitochondrial function in normal and immune cells. The therapeutic challenge is therefore to exploit the coupling between mitochondrial metabolism and apoptotic dependence selectively in malignant cells.

### 5.4. Combining Mitochondrial Targeting with Immunotherapy

Mitochondrial interventions may alter the cancer-immune interaction in two potentially complementary ways. First, selective disruption of tumor mitochondrial fitness may reduce the capacity of malignant cells to withstand oxidative stress, nutrient limitation, apoptosis, and immune-mediated killing. Second, interventions that preserve immune-cell mitochondrial function may improve T-cell persistence and cytotoxicity. However, this dual rationale creates a major therapeutic challenge. A mitochondrial inhibitor that weakens cancer cells may also reduce the respiratory capacity and functional resilience of T cells, natural killer cells, dendritic cells, and other immune populations. Therefore, the rationale for combining mitochondrial agents with immune checkpoint inhibitors, CAR-T cells, or bispecific antibodies must be evaluated according to the specific mechanism, dose, schedule, and target cell population. The therapeutic consequences of mitochondrial targeting may therefore depend critically on the cellular compartment affected, the magnitude and duration of mitochondrial perturbation, and the timing of treatment relative to immunotherapy.

Potential combination strategies include transient tumor-directed OXPHOS inhibition before immune therapy, selective inhibition of tumor-specific anti-apoptotic pathways, metabolic conditioning of cellular products before infusion, and the use of agents that limit tumor oxidative adaptation without suppressing immune-cell activation.

Mitochondrial stress in tumor cells may also influence immunogenicity through the release of mitochondrial DNA and other danger-associated signals, potentially activating innate immune pathways such as cGAS–STING and enhancing antitumor immune responses [85]. However, these effects are context-dependent, and excessive or persistent mitochondrial stress may instead promote immune dysfunction or tumor adaptation.

Accordingly, mitochondrial targeting should not be considered intrinsically immunostimulatory. Tumor-selective mitochondrial perturbation may increase immune visibility, whereas mitochondrial damage in immune effector cells may impair persistence and cytotoxic function. This bidirectional effect is central to the mitochondrial functional checkpoint proposed in this review.

An additional therapeutic opportunity involves selective induction of mitochondrial stress in tumor cells to promote mitochondrial DNA release and activation of innate immune sensing pathways. Tumor-directed mitochondrial injury may activate the cyclic GMP-AMP synthase-stimulator of interferon genes axis, promote immunogenic cell death, improve dendritic-cell activation, and increase the immunological visibility of cancer cells. However, this strategy requires careful cellular targeting because mitochondrial dysfunction in T cells, natural killer cells, or dendritic cells may instead impair antitumor immunity. The therapeutic objective is therefore to induce immunogenic mitochondrial stress selectively in malignant cells while preserving mitochondrial integrity in immune effectors [86,87]. Supporting this concept, a recent study in AML demonstrated that inhibition of ADSS2-mediated de novo AMP biosynthesis impaired mitochondrial respiration and restored sensitivity to the BH3 mimetic venetoclax, providing proof-of-concept that indirect metabolic targeting can overcome mitochondrial-dependent therapeutic resistance [55]. Although this study did not directly evaluate immunotherapy, it illustrates how metabolic manipulation of tumor mitochondria can alter apoptotic vulnerability and provides a rationale for exploring complementary combinations with immune-based therapies.

Overall, the most promising strategy may therefore be to uncouple tumor mitochondrial vulnerability from immune-cell mitochondrial dependence: weaken mitochondrial fitness in malignant cells while preserving or enhancing mitochondrial resilience in antitumor immune effectors. This approach remains largely preclinical and requires prospective evaluation of both antitumor efficacy and immune-cell function.

These approaches remain investigational and should be framed cautiously until supported by prospective clinical evidence.

**Figure 4:**
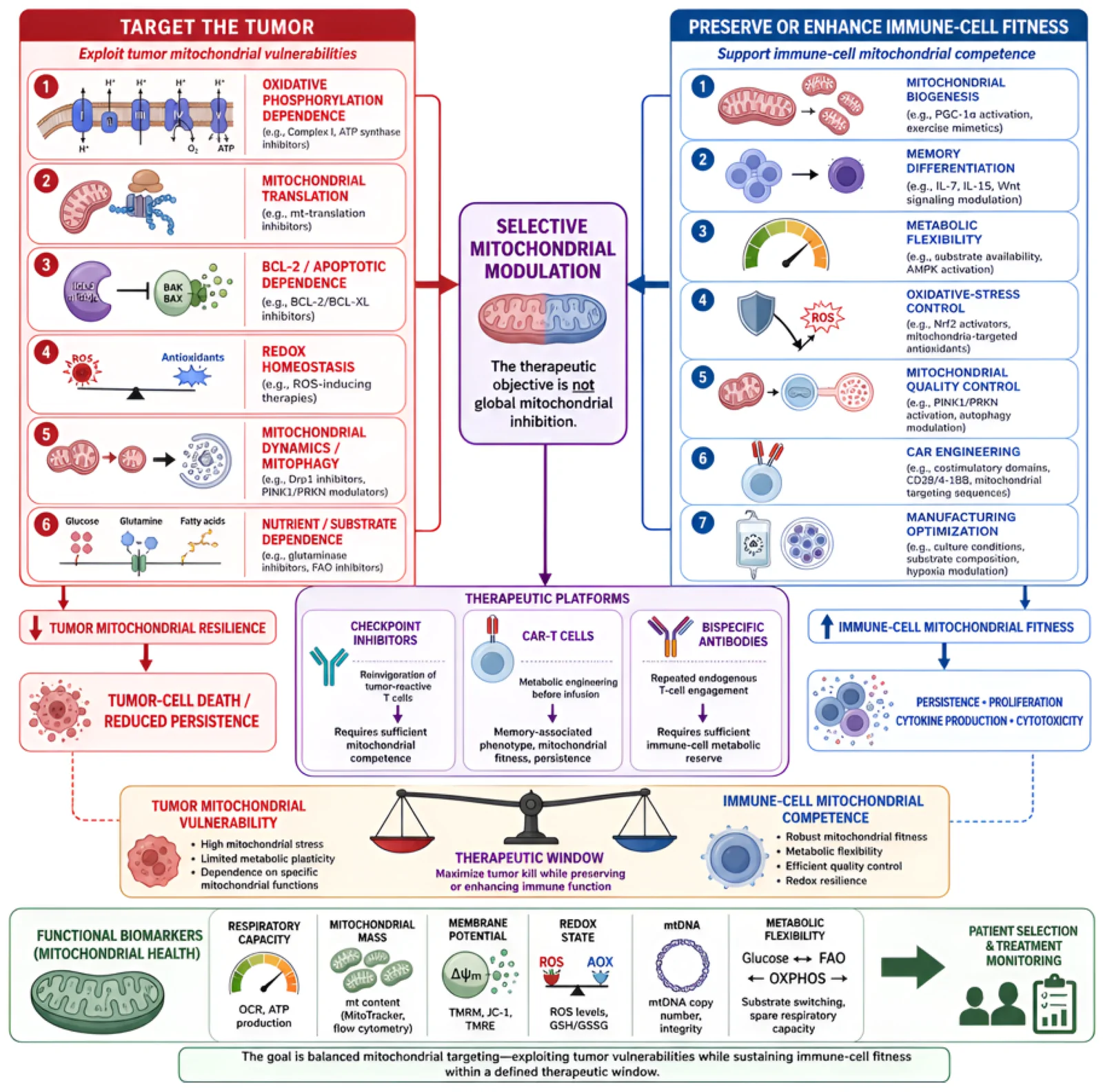
Therapeutic strategies targeting the mitochondrial functional immune checkpoint. Mitochondrial vulnerabilities can be targeted through modulation of oxidative metabolism, mitochondrial dynamics and quality control, redox pathways, apoptosis, mitochondrial transfer, or metabolic dependencies. Therapeutic strategies should selectively weaken tumor-cell mitochondrial adaptation while preserving or enhancing the mitochondrial competence of immune effector cells. The figure summarizes potential combination strategies and their principal translational challenges.

The therapeutic strategies targeting the mitochondrial functional checkpoint, including approaches directed at tumor-cell mitochondrial vulnerabilities and strategies designed to preserve or enhance immune-cell mitochondrial competence, are summarized in Figure 4.

Future strategies will likely require a dual approach that simultaneously weakens tumor mitochondrial adaptation and preserves or restores immune-cell mitochondrial fitness.

### 5.5. Mitochondrial Engineering of Immune-Cell Therapies

Adoptive cellular therapies offer a distinctive opportunity because the metabolic state of therapeutic cells can be influenced before infusion. T cells may be selected, expanded, conditioned, or genetically engineered to favor memory-associated differentiation, enhanced mitochondrial fitness, and resistance to exhaustion. Less differentiated T-cell populations, including stem-cell memory and central-memory subsets, generally display improved proliferative capacity and long-term persistence. These populations are characterized by greater mitochondrial reserve and oxidative metabolic flexibility than terminally differentiated effector populations [88]. CAR design can also affect metabolic programming. CARs incorporating 4-1BB costimulatory domains can promote mitochondrial biogenesis, fatty-acid oxidation, respiratory capacity, and memory-associated differentiation, whereas CD28-based CARs often induce stronger glycolytic activation and more rapid effector differentiation [77]. These differences do not establish that one costimulatory domain is universally superior; rather, they illustrate that CAR architecture can shape the metabolic state and functional persistence of therapeutic T cells.

Additional approaches under investigation include modulation of cytokine exposure during manufacturing, nutrient optimization, reduction in tonic CAR signaling, control of oxidative stress, and genetic enhancement of pathways that support mitochondrial quality control and metabolic flexibility.

Recent experimental studies have provided proof-of-concept that mitochondrial fitness can itself be therapeutically engineered. For example, SLC25A51 mRNA–lipid nanoparticle engineering increased mitochondrial respiratory function and ATP production in T cells and enhanced antitumor activity in preclinical models, including CAR-T cells [89]. Other emerging strategies target mitochondrial dynamics, substrate utilization, redox homeostasis, and metabolic signaling to preserve CAR-T-cell function under prolonged tumor-associated stress [90].

These strategies may be particularly important for solid tumors, where engineered T cells encounter hypoxia, nutrient scarcity, stromal barriers, and prolonged antigenic stimulation. However, mitochondrial engineering should not be equated simply with increasing oxidative phosphorylation. The therapeutic objective is to preserve mitochondrial quality, reserve, and metabolic flexibility while avoiding excessive mitochondrial stress or inappropriate activation. This distinction is particularly relevant to the mitochondrial functional checkpoint proposed in this review, because interventions that enhance immune-cell mitochondrial resilience could potentially be combined with tumor-directed mitochondrial vulnerabilities to widen the therapeutic window.

At present, these approaches remain largely investigational and are supported predominantly by preclinical evidence. Their clinical translation will require demonstration that engineered mitochondrial fitness improves persistence and antitumor efficacy without increasing exhaustion, toxicity, or unwanted effects on immune-cell function.

### 5.6. Hematological Malignancies as a Focused Translational Model for Mitochondrial Targeting

Hematological malignancies provide a particularly attractive setting for mitochondrial-directed therapy because malignant cells are often accessible for serial sampling and functional metabolic analysis. In acute myeloid leukemia, the mitochondrial dependence of leukemia stem cells and the clinical activity of venetoclax-based combinations provide a strong translational foundation for further mitochondrial targeting. The bone-marrow niche provides a particularly relevant example of microenvironment-mediated mitochondrial support. Bone-marrow stromal cells can transfer mitochondria to leukemic cells, thereby enhancing oxidative phosphorylation, ATP production, redox buffering, and survival during chemotherapy exposure. In acute myeloid leukemia, NADPH oxidase 2-derived oxidative stress has been implicated in promoting stromal-to-leukemia mitochondrial transfer, suggesting that leukemic cells may actively solicit mitochondrial support from the microenvironment. These observations support the rationale for therapeutic strategies that target not only leukemia-cell-intrinsic oxidative metabolism but also the intercellular mechanisms that restore mitochondrial fitness after treatment-induced stress [25,91].

In AML, leukemia-derived NADPH oxidase 2 (NOX2)-dependent oxidative stress has been shown to stimulate mitochondrial transfer from bone-marrow stromal cells through tumor-derived tunneling nanotubes. Inhibition of NOX2 reduced mitochondrial transfer, increased AML apoptosis, and improved survival in preclinical models [51,92]. These observations suggest that the bone-marrow microenvironment can function not only as a source of trophic signals but also as a source of mitochondrial support that helps leukemia cells restore bioenergetic capacity after therapeutic stress. Targeting the mechanisms that enable mitochondrial transfer may therefore complement strategies directed against leukemia-cell-intrinsic oxidative metabolism.

In multiple myeloma, mitochondrial metabolism is closely associated with immunoglobulin production, endoplasmic-reticulum stress, proteostasis, redox regulation, and resistance to therapy [12].

Myeloma cells can also acquire functional mitochondria from bone-marrow stromal cells, with mitochondrial transfer supporting oxidative phosphorylation and cellular survival [93]. Metabolic adaptation to proteasome inhibition involves coordinated changes in mitochondrial metabolism, lipid utilization, redox homeostasis, and amino-acid availability, generating metabolic dependencies that may become therapeutically exploitable during adaptation to proteotoxic stress [94].

These features provide a rationale for investigating mitochondrial-directed approaches in combination with established anti-myeloma therapies, including proteasome inhibitors and immunotherapies. However, combinations with CAR-T cells or bispecific antibodies remain largely investigational, and their potential benefit will depend on whether tumor mitochondrial vulnerabilities can be exploited without compromising the metabolic fitness of immune effector cells. The key principle is that mitochondrial targeting should not be applied as a uniform strategy across all hematological cancers. Instead, it should be guided by the metabolic phenotype of the tumor, the state of the immune compartment, the contribution of the bone-marrow microenvironment, and the anticipated interaction with concurrent therapies.

A summary of representative mitochondrial-targeting and immune-modulatory interventions, including their molecular targets, disease and experimental context, evidence level, therapeutic outcomes, and principal translational limitations, is provided in Table 1.

### 5.7. Challenges and Future Directions

Several barriers must be addressed before mitochondrial targeting can be broadly integrated into oncology practice. First, mitochondrial dependency is heterogeneous. Tumors may rely on different substrates, respiratory pathways, antioxidant systems, or anti-apoptotic proteins. A biomarker-guided approach will therefore be necessary. Second, mitochondrial function is dynamic. Treatment exposure can induce metabolic rewiring, allowing tumors to bypass a targeted pathway. Longitudinal metabolic monitoring may be required to identify emerging resistance mechanisms. Third, mitochondria are essential for normal tissues and antitumor immune cells. Mitochondrial transfer introduces an additional layer of complexity because its consequences may depend on the direction of transfer and the identity of both donor and recipient cells.

This heterogeneity also limits direct extrapolation between hematological and solid malignancies. Shared mitochondrial principles should therefore be distinguished from disease-specific dependencies: a pathway that represents a therapeutic vulnerability in one disease may serve a different adaptive function or have a different therapeutic window in another. Comparative studies across tumor classes will be important to identify which components of mitochondrial fitness represent general oncologic mechanisms and which remain lineage- or context-specific.

Blocking stromal-to-tumor mitochondrial transfer could limit metabolic rescue and treatment resistance, whereas indiscriminate inhibition of mitochondrial exchange could impair mitochondrial support of cytotoxic lymphocytes or other immune populations. Conversely, therapeutic mitochondrial augmentation may improve T-cell persistence but could be harmful if delivered to malignant cells or immunosuppressive populations. Future approaches should therefore prioritize context-selective modulation based on donor–recipient identity, cargo quality, and recipient-specific mitochondrial vulnerability [26]. The therapeutic window of any mitochondrial intervention will depend on its selectivity, schedule, combination partners, and capacity to spare immune-cell function. Finally, clinical translation will require functional biomarkers that measure more than mitochondrial gene expression. Respiratory capacity, mitochondrial mass, membrane potential, redox state, metabolic substrate use, and stress-response signatures may ultimately help identify patients whose tumors are vulnerable to mitochondrial intervention and whose immune cells can sustain immunotherapy. Importantly, no single parameter is likely to define mitochondrial fitness across tumor and immune-cell populations. Multidimensional and longitudinal profiling will therefore probably be required to distinguish adaptive mitochondrial states from irreversible dysfunction and to identify therapeutically actionable vulnerabilities.

Longer-term approaches, including mitochondria-based cancer vaccines, precision mitochondrial-genome editing, and engineered mitochondrial transfer, remain early-stage strategies and should currently be regarded as exploratory rather than clinically actionable approaches [24]. Nevertheless, they illustrate the broader possibility of manipulating mitochondrial signaling, organelle quality, and metabolic fitness with increasing cellular specificity.

The ultimate objective is therefore not indiscriminate mitochondrial inhibition, but selective modulation of mitochondrial fitness. Future therapeutic strategies will need to weaken the metabolic adaptability of tumor cells while simultaneously preserving or enhancing the mitochondrial resilience, and metabolic flexibility of immune effectors, thereby expanding the therapeutic window of metabolism-based immunotherapy.

**Figure 5:**
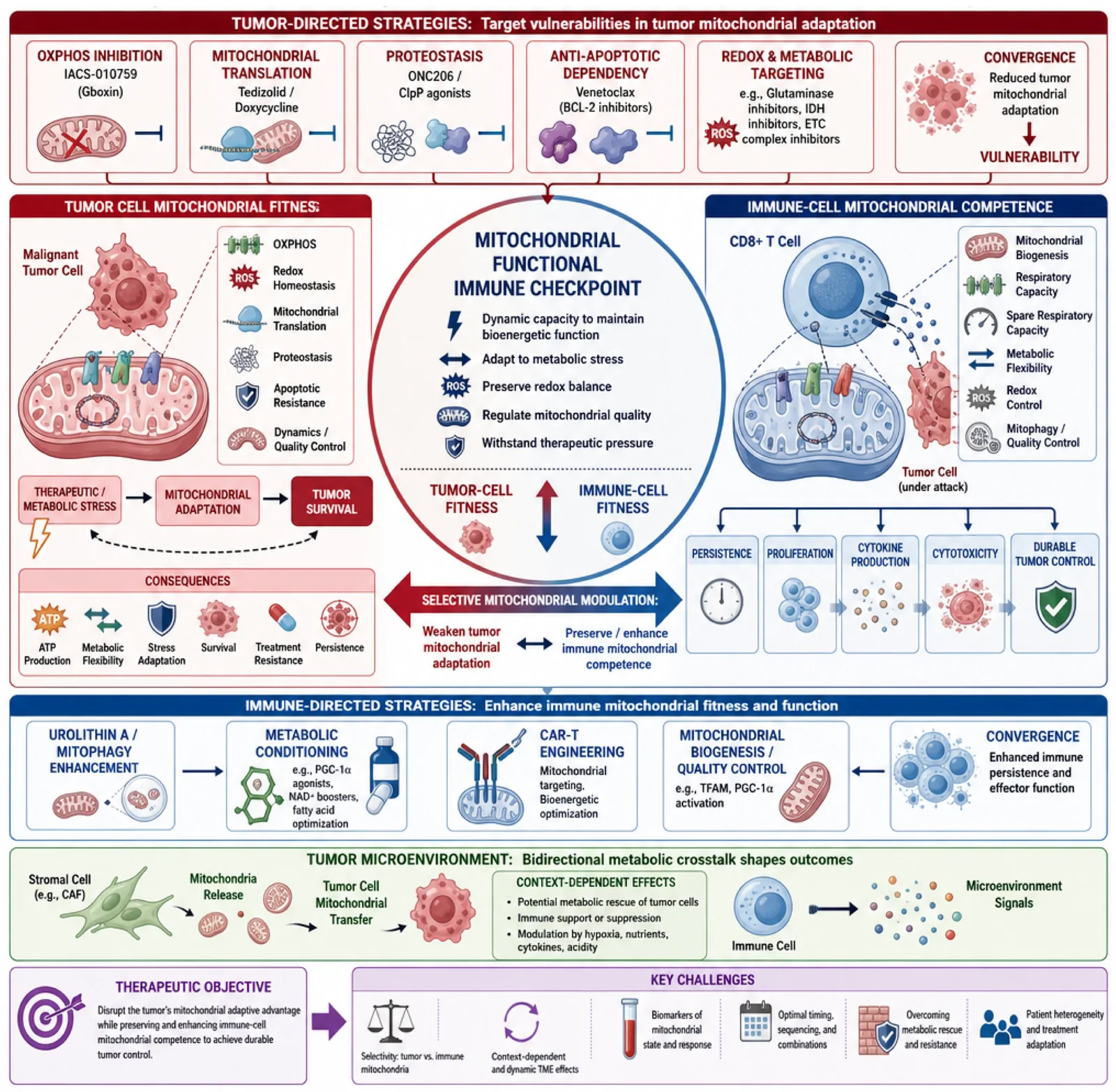
Therapeutic modulation of the mitochondrial functional checkpoint. Schematic overview of representative strategies targeting distinct components of mitochondrial biology in tumor cells and immune cells. Tumor-directed approaches include inhibition of oxidative phosphorylation, mitochondrial translation, and mitochondrial proteostasis, whereas immune-directed strategies may enhance mitochondrial quality control and metabolic competence. The figure emphasizes that mitochondrial interventions can have opposing effects depending on the cellular target and biological context. Strategies that suppress tumor mitochondrial adaptation may enhance tumor vulnerability, whereas preservation or enhancement of immune-cell mitochondrial fitness may support durable antitumor responses. Most approaches remain investigational, and their therapeutic feasibility will depend on cellular selectivity, treatment context, combination strategy, and preservation of immune-cell function.

Representative therapeutic strategies targeting distinct components of the mitochondrial functional checkpoint are summarized in Figure 5.

## 6. Mitochondrial Biomarkers and the Future of Functional Precision Immuno-Oncology

Precision oncology has traditionally focused on identifying genomic alterations that can be matched to targeted therapies. In immuno-oncology, predictive strategies have expanded to include PD-L1 expression, tumor mutational burden, microsatellite instability, immune-cell infiltration, gene-expression signatures, and measurable residual disease. These biomarkers provide important information, but they do not fully capture the dynamic functional state of tumor cells or immune populations. A tumor may express a target antigen and contain infiltrating lymphocytes while remaining resistant to immunotherapy because malignant cells are metabolically adaptable or immune cells are metabolically dysfunctional. Mitochondrial profiling may therefore provide a complementary functional layer of information. Rather than assessing only whether immune cells are present or whether a tumor is antigenic, mitochondrial biomarkers may help determine whether tumor and immune compartments retain the metabolic capacity required for a sustained therapeutic response. Current immunotherapy biomarkers have substantial but incomplete predictive value, emphasizing the need for integrated models that include genomic, immune, clinical, and functional features [20,95].

Mitochondrial biomarkers should not be viewed as replacements for established genomic or immune biomarkers. Their potential value lies in adding a functional dimension that captures the ability of tumor and immune cells to respond to metabolic, oxidative, and therapeutic stress. Such information may be particularly relevant when conventional biomarkers identify an apparently favorable immune contexture but fail to explain primary resistance or subsequent loss of response.

### 6.1. Functional Mitochondrial Profiling

Mitochondrial biomarkers should include direct functional measurements whenever feasible. Potential parameters include oxygen-consumption rate, basal and maximal respiratory capacity, spare respiratory capacity, mitochondrial membrane potential, mitochondrial mass, reactive oxygen species production, mitochondrial DNA copy number, and expression of genes associated with mitochondrial biogenesis, oxidative phosphorylation, mitophagy, and the integrated stress response. Among these measures, spare respiratory capacity may be particularly relevant for immune-cell function. It reflects the ability of a cell to increase mitochondrial respiration in response to stress or increased energy demand. Memory-associated T cells generally demonstrate greater spare respiratory capacity than terminally differentiated effector populations, whereas exhausted tumor-infiltrating T cells may show reduced mitochondrial biogenesis and impaired metabolic flexibility [18,68]. Markers of mitochondrial biogenesis may also be informative. The expression of peroxisome proliferator-activated receptor gamma coactivator-1α, mitochondrial transcription factor A, respiratory-chain genes, and fatty-acid-oxidation programs may help characterize whether immune cells retain the capacity to sustain oxidative metabolism. However, transcriptomic markers should not be assumed to provide a direct substitute for functional metabolic assays. Gene-expression profiles can suggest mitochondrial states, but they do not necessarily measure actual respiratory performance.

Recent clinical-translational evidence further illustrates this distinction. Mitochondrial membrane potential in circulating CD8^+^ T cells was associated with both oxidative-phosphorylation and exhaustion-related programs during cancer immunotherapy, indicating that a single mitochondrial parameter cannot be interpreted as a universal surrogate of immune fitness [96]. Accordingly, mitochondrial fitness should be considered a multidimensional phenotype encompassing respiratory reserve, metabolic flexibility, redox control, mitochondrial quality, and the capacity to sustain effector function under stress.

For mitochondrial-transfer biology, detection of donor-derived mitochondrial material alone should not be considered sufficient evidence of functional relevance. Biomarker studies should distinguish transient uptake from durable integration by assessing mitochondrial membrane potential, respiratory capacity, mitochondrial DNA integrity, mitophagy susceptibility, fusion with the host mitochondrial network, and persistence through cell division. These parameters may help identify tumors in which mitochondrial transfer is a biologically meaningful contributor to resistance or immune dysfunction rather than an incidental consequence of cellular stress [25]. Mitochondrial stress signatures may provide an additional dimension. Alterations in reactive oxygen species, mitophagy pathways, mitochondrial unfolded-protein-response signaling, membrane potential, or mitochondrial DNA release may identify cells undergoing metabolic adaptation, treatment-induced stress, or progressive exhaustion. Their interpretation will require careful attention to cellular context because the same stress pathway may be adaptive in one setting and detrimental in another.

The clinical implementation of these assays will require standardized sample handling, appropriate cellular resolution, and harmonized definitions of mitochondrial fitness. Longitudinal measurements may be particularly informative because mitochondrial states can change during treatment and may identify emerging resistance before overt clinical progression.

### 6.2. Mitochondrial DNA and Tumor Evolution

Mitochondrial DNA is increasingly recognized as a potentially informative component of cancer biology. Changes in mitochondrial DNA copy number, heteroplasmy, mutations, and mitochondrial genome integrity may influence oxidative metabolism, redox balance, cellular stress responses, and clonal evolution. In addition, mitochondrial DNA released from damaged mitochondria can activate innate immune pathways, linking mitochondrial stress to inflammatory signaling [21]. At present, mitochondrial DNA alterations should be regarded as promising but incompletely validated biomarkers. Their biological and clinical significance varies by tumor type, sample source, disease stage, and coexisting nuclear genomic alterations. Prospective studies should determine whether mitochondrial DNA features add predictive value beyond established genomic and immunological biomarkers.

Mitochondrial DNA may also provide information that is distinct from nuclear genomic profiling because heteroplasmy and mitochondrial genome alterations can vary between malignant subclones and may change under therapeutic selection. However, the interpretation of mtDNA alterations requires careful distinction between functional driver events, passenger mutations, and changes related to mitochondrial stress or clonal selection.

In the context of immunotherapy, mtDNA is particularly relevant because mitochondrial damage can generate cytosolic DNA and activate cGAS–STING and related innate immune pathways [21,59]. Thus, mtDNA profiling may eventually provide information not only about tumor evolution but also about the immunogenic consequences of mitochondrial dysfunction. Whether these alterations can reliably predict treatment response remains uncertain and requires prospective validation.

### 6.3. Single-Cell and Spatial Approaches to Mitochondrial Heterogeneity

Bulk tumor profiling can obscure the metabolic diversity of malignant cells, stromal populations, and immune-cell subsets. Single-cell technologies offer the opportunity to identify distinct transcriptional and metabolic states within the same tumor, including populations characterized by oxidative stress, mitochondrial biogenesis, hypoxia adaptation, or exhaustion-associated programs. However, mitochondrial gene expression in single-cell RNA sequencing should be interpreted cautiously. A high proportion of mitochondrial transcripts may indicate cellular stress or reduced viability during sample processing, rather than a biologically meaningful increase in mitochondrial activity. Therefore, mitochondrial transcript abundance should be integrated with cell-state markers, protein measurements, metabolic assays, and spatial context rather than used alone as a surrogate for mitochondrial fitness. Spatial transcriptomic, proteomic, and imaging approaches may be particularly informative because metabolic states are strongly shaped by local conditions. Tumor cells and immune populations near necrotic, hypoxic, or nutrient-depleted regions may display mitochondrial programs that differ substantially from those in vascularized tumor areas, invasive margins, or lymphoid aggregates. The integration of single-cell and spatial approaches with functional mitochondrial measurements may therefore be particularly valuable for defining the mitochondrial functional checkpoint at cellular resolution. Such approaches could identify tumor–immune neighborhoods in which mitochondrial stress, metabolic competition, mitochondrial transfer, and immune dysfunction converge, potentially revealing vulnerabilities that are obscured by bulk profiling.

Nevertheless, most currently available single-cell approaches infer mitochondrial function indirectly. Future studies should therefore prioritize multimodal strategies that combine mitochondrial transcriptomics with protein-level measurements, functional respiration or redox assays, spatial localization, and longitudinal clinical sampling.

### 6.4. Mitochondrial Profiling and Measurable Residual Disease

Measurable residual disease has transformed response assessment and risk stratification in hematological malignancies. In acute myeloid leukemia, measurable residual disease is strongly associated with relapse risk and survival outcomes [97]. In multiple myeloma, sensitive measurable-residual-disease assessment has become an important component of disease evaluation and clinical-trial design [98]. However, MRD primarily measures the burden of persistent disease and provides limited information about the functional state of residual malignant cells or the immune compartment.

A residual population characterized by oxidative metabolic flexibility, enhanced stress tolerance, apoptosis resistance, or stem-like properties could theoretically have a different relapse potential from an MRD population of similar size but lower adaptive capacity. Similarly, the functional state of immune cells may influence whether residual disease remains controlled after treatment. Integrating MRD with mitochondrial profiling could therefore provide complementary information on both residual disease burden and the biological fitness of the cells that persist after therapy.

Potential mitochondrial measurements could include respiratory capacity, mitochondrial mass, membrane potential, redox state, mitochondrial DNA alterations, and stress-response signatures. Such measurements might help distinguish quantitatively similar MRD states according to their metabolic or functional characteristics. However, this remains a hypothesis-generating approach. No mitochondrial parameter has yet been validated as an independent predictor of relapse beyond established MRD measurements, and mitochondrial profiling should not currently be used to modify treatment decisions outside appropriately designed prospective studies.

Future studies should determine whether mitochondrial features measured at MRD-positive or MRD-negative time points provide incremental prognostic information and whether longitudinal changes in mitochondrial fitness can identify emerging metabolic adaptation before overt clinical relapse.

### 6.5. Mitochondrial Biomarkers in CAR-T-Cell and Bispecific-Antibody Therapy

Cellular therapy provides a practical setting for mitochondrial characterization because the therapeutic product can be evaluated before infusion and monitored longitudinally after treatment. Candidate biomarkers in CAR-T-cell therapy include mitochondrial mass, respiratory capacity, spare respiratory capacity, oxidative-stress markers, differentiation state, memory-associated metabolic programs, and exhaustion-associated transcriptional signatures. Clinical response to CD19-directed CAR-T-cell therapy in chronic lymphocytic leukemia has been associated with features of the infused product consistent with memory differentiation and metabolic fitness [78]. These observations support the concept that functional characterization of the cellular product may complement conventional measures such as viability, transduction efficiency, cell dose, and immunophenotype.

Nevertheless, mitochondrial fitness should not be considered an isolated determinant of CAR-T-cell efficacy.

CAR design, T-cell differentiation state, tonic signaling, antigen exposure, cytokine environment, tumor burden, and the tumor microenvironment all influence CAR-T-cell persistence and function. Recent experimental evidence further indicates that intracellular signaling networks can modify CAR-T-cell fitness in the context of tumor immune escape. In models of CD58-loss-driven resistance, DUSP6 ablation restored CAR-T-cell activity through enhancement of AP-1 signaling, improving proliferation, cytokine production, and antitumor function [98]. Although this study does not directly establish mitochondrial dysfunction as the mechanism of resistance, it illustrates the broader principle that the functional state of therapeutic T cells can be actively modified and may represent a complementary target to tumor-directed interventions.

In bispecific-antibody therapy, the relevant immune compartment is the patient’s endogenous T-cell population. Baseline T-cell number, differentiation state, previous antigen exposure, exhaustion phenotype, and treatment history may influence the capacity to sustain repeated tumor-cell engagement. Mitochondrial measurements could potentially complement these parameters by identifying functional limitations in the metabolic reserve of endogenous T cells. However, the specific contribution of mitochondrial fitness to response or resistance to bispecific antibodies remains incompletely defined in clinical studies. Accordingly, mitochondrial profiling in this setting should currently be considered an investigational biomarker strategy rather than an established predictor of treatment outcome. Prospective studies should determine whether mitochondrial features independently predict response, duration of benefit, toxicity, or loss of response and whether they provide information beyond conventional immune-cell phenotyping.

### 6.6. From Precision Oncology to Functional Precision Immuno-Oncology

The next phase of precision oncology will likely require integration of static molecular information with dynamic functional measurements. Genomic data identify mutations, copy-number alterations, and targetable pathways. Immune profiling identifies infiltrating cell populations and inhibitory mechanisms.

Current mitochondrial biomarkers also have important limitations. Mitochondrial gene expression, mitochondrial DNA alterations, protein abundance, mitochondrial mass, membrane potential, and respiratory measurements capture different dimensions of mitochondrial biology and should not be considered interchangeable surrogates of mitochondrial fitness. Their interpretation is further complicated by intratumoral and intercellular heterogeneity, differences in sample processing, tissue-specific mitochondrial phenotypes, lack of standardized analytical platforms and thresholds, and dynamic changes induced by treatment. In bulk tumor samples, mitochondrial signals may also reflect malignant, stromal, or immune-cell populations, making attribution to a specific cellular compartment challenging. Moreover, most candidate mitochondrial biomarkers remain insufficiently validated against prospective clinical outcomes. Functional assays assessing respiratory capacity, spare respiratory capacity, substrate utilization, redox state, or adaptive responses to metabolic stress may therefore provide more informative measures of mitochondrial fitness than static molecular markers alone. Standardization, longitudinal assessment, and prospective validation will be required before mitochondrial biomarkers can be incorporated reliably into clinical decision-making.

Mitochondrial profiling could add a complementary functional dimension by assessing the capacity of malignant cells to adapt to stress and the ability of immune cells to sustain antitumor activity.

Potential measurements include mitochondrial respiration, spare respiratory capacity, mitochondrial mass, membrane potential, redox state, mitochondrial DNA features, mitochondrial dynamics, and stress-response signatures. Their clinical interpretation, however, will require standardized assays, reproducible sample processing, disease-specific reference ranges, longitudinal validation, and demonstration of incremental clinical value over established biomarkers. Artificial-intelligence and machine-learning approaches may eventually facilitate the integration of high-dimensional genomic, transcriptomic, proteomic, metabolic, imaging, and clinical datasets. However, predictive models will need to be interpretable, externally validated, and clinically actionable before they can be incorporated into routine practice.

Mitochondrial biomarkers should therefore be viewed as complementary rather than replacement measures. Their potential value may include identifying metabolically resilient tumors, characterizing immune-cell functional competence, detecting treatment-associated metabolic adaptation, refining selection for cellular or T-cell-redirecting therapies, and informing rational combinations that target tumor mitochondrial dependencies while preserving immune-cell fitness. In hematological malignancies, integration with MRD assessment may be particularly attractive because MRD quantifies residual disease burden, whereas functional mitochondrial measurements could potentially characterize the biological state of the residual clone and the immune compartment.

The potential clinical applications of mitochondrial profiling within functional precision immuno-oncology are summarized in Table 2. At present, these applications remain investigational and should not be interpreted as evidence that mitochondrial profiling is ready for routine treatment selection. Rather, the emerging framework suggests that mitochondrial features could eventually complement genomic, immunological, and clinical biomarkers by providing dynamic information on the adaptive capacity of malignant and immune cells.

The ultimate goal of functional precision immuno-oncology is therefore not to replace established biomarkers but to integrate molecular identity with functional state. Such an approach could help identify metabolically resilient disease, characterize immune-cell competence, detect mechanisms of treatment persistence or resistance, and guide combination strategies that selectively exploit tumor mitochondrial vulnerabilities while preserving or enhancing immune-cell fitness.

## 7. Discussion

The traditional view of mitochondria as passive energy-producing organelles is no longer sufficient to explain their role in cancer biology. Mitochondria regulate oxidative metabolism, redox homeostasis, biosynthesis, apoptotic signaling, stress adaptation, inflammatory responses, and cell-state plasticity. Through these interconnected functions, they influence how malignant cells evolve, survive therapy, interact with their micro-environment, and evade immune-mediated elimination [1,8]. The relevance of these functions, however, is highly context-dependent: mitochondrial activity is not synonymous with mitochondrial fitness, and high oxidative phosphorylation is neither necessary nor sufficient to define a metabolically fit cancer cell. Rather, mitochondrial fitness should be understood as the capacity of a cell to maintain or restore mitochondrial competence while adapting to metabolic, environmental, immune, and therapeutic selective pressures.

This distinction is particularly important because a highly glycolytic tumor may remain metabolically resilient if it can flexibly switch substrates or pathways, whereas an immune cell with substantial respiratory activity may nevertheless be functionally compromised if it cannot maintain mitochondrial homeostasis during chronic stimulation.

In this context, we propose the mitochondrial functional immune checkpoint as a conceptual model linking two complementary dimensions of mitochondrial biology. In malignant cells, mitochondrial plasticity can support adaptation to hypoxia, nutrient limitation, oxidative stress, immune attack, and anticancer treatment through changes in substrate utilization, respiratory activity, mitochondrial dynamics, quality control, redox balance, and apoptotic thresholds [27,30]. These adaptations may favor the persistence of slow-cycling, stem-like, metastatic, or therapy-resistant populations. Conversely, immune effector cells require sufficient mitochondrial resilience and metabolic flexibility to sustain proliferation, migration, cytokine production, and cytotoxicity within the tumor microenvironment. Tumor-infiltrating T cells are exposed to chronic antigen stimulation, hypoxia, nutrient competition, lactate accumulation, and oxidative stress, conditions that can impair mitochondrial biogenesis and respiratory capacity and contribute to exhaustion-associated dysfunction [17,66]. The proposed checkpoint therefore does not represent a new receptor–ligand pathway. Rather, it describes a functional constraint on immune surveillance imposed by the mitochondrial state of both malignant and immune cells.

This distinction also helps define the conceptual boundaries of this model. Mitochondrial plasticity refers to the ability of mitochondria and mitochondrial networks to remodel in response to changing conditions; mitochondrial quality control encompasses mechanisms such as biogenesis, fusion, fission, and mitophagy; and mitochondrial fitness refers to the resulting functional capacity to maintain or recover mitochondrial competence under selective pressure. The mitochondrial functional immune checkpoint extends these concepts to the tumor–immune interface by considering their reciprocal consequences for immune elimination. This concept is therefore best viewed as an integrative model rather than as a newly established biological entity. Its value will ultimately depend on whether it generates experimentally testable hypotheses and clinically useful biomarkers beyond those provided by conventional measures of metabolism or immune activation.

The tumor–immune interface provides several mechanisms through which mitochondrial fitness may influence therapeutic response. Tumor-cell mitochondrial adaptation can alter oxygen consumption, nutrient utilization, redox balance, metabolite availability, and innate immune signaling, thereby reshaping the metabolic conditions encountered by infiltrating immune cells. Conversely, immune-cell mitochondrial dysfunction may limit the ability of activated T cells to sustain antitumor activity even when antigen recognition and proximal receptor signaling remain intact. Mitochondrial stress and mtDNA release can additionally influence innate immune pathways, including cGAS–STING signaling, although the consequences are context-dependent and may differ according to the intensity and duration of mitochondrial damage. Similarly, intercellular mitochondrial transfer may modify tumor or immune-cell fitness, but its frequency, directionality, durability, and clinical relevance remain incompletely defined. These mechanisms should therefore be regarded as interconnected but not interchangeable components of this integrative model.

The implications are particularly relevant to contemporary immunotherapy. Immune checkpoint inhibitors can reinvigorate selected tumor-reactive T-cell populations, particularly progenitor-like exhausted cells that retain proliferative capacity, but they may be less effective when immune dysfunction has progressed toward severe mitochondrial impairment or terminal exhaustion. CAR-T-cell therapy provides a distinct opportunity because mitochondrial fitness can be influenced during manufacturing through T-cell selection, CAR design, cytokine conditioning, metabolic optimization, and genetic engineering. CARs incorporating 4-1BB costimulatory domains, for example, can favor memory-associated differentiation and mitochondrial programs associated with persistence, although neither 4-1BB nor CD28 can be considered universally superior across clinical contexts [74,75]. Bispecific antibodies, in contrast, depend on the patient’s endogenous immune repertoire, making their efficacy potentially sensitive to the pre-existing metabolic and mitochondrial competence of T cells. Nevertheless, the specific contribution of mitochondrial dysfunction to resistance to bispecific antibodies remains insufficiently defined clinically and should currently be regarded as a testable hypothesis rather than an established mechanism [76,77].

These considerations have important therapeutic implications. The goal of mitochondrial targeting should not be indiscriminate inhibition of mitochondrial respiration. Mitochondria are essential for normal tissues and for the immune cells required to achieve antitumor responses. The clinically relevant challenge is therefore to identify tumor-selective mitochondrial dependencies while preserving, restoring, or engineering mitochondrial competence in immune effectors. Venetoclax-based therapy in AML provides an important example of how mitochondrial apoptotic dependence can be therapeutically exploited, while emerging resistance mechanisms illustrate how metabolic adaptation can limit treatment durability [52,87]. More broadly, mitochondrial-directed strategies targeting oxidative phosphorylation, mitochondrial translation, redox regulation, apoptosis, mitochondrial dynamics, quality control, or metabolic dependencies remain heterogeneous in their degree of clinical development. Their feasibility will depend on therapeutic selectivity, treatment schedule, combination partners, and the ability to avoid compromising immune-cell function.

A further challenge is that mitochondrial vulnerabilities are dynamic rather than fixed. Treatment pressure can induce metabolic rewiring, allowing tumor cells to bypass an inhibited pathway or obtain mitochondrial support from the microenvironment. Mitochondrial transfer illustrates this complexity particularly well: blocking stromal-to-tumor transfer might reduce metabolic rescue, whereas indiscriminate inhibition of mitochondrial exchange could potentially impair beneficial support of immune cells. Similarly, interventions designed to enhance mitochondrial function in T cells could theoretically benefit malignant cells if their effects are not sufficiently cell-selective. These opposing possibilities argue against a universal “more mitochondria” or “less mitochondria” therapeutic strategy and instead favor context-selective manipulation of mitochondrial fitness.

The biomarker implications are equally important. Conventional genomic biomarkers, PD-L1 expression, tumor mutational burden, immune-cell infiltration, and measurable residual disease provide valuable information but do not fully capture the dynamic functional state of either malignant or immune cells. Mitochondrial profiling could potentially complement these measures by capturing the functional state and adaptive capacity of malignant and immune cells [94,95]. In hematological malignancies, integration of mitochondrial profiling with measurable residual disease could be particularly informative because residual disease burden and the functional state of the surviving clone represent complementary dimensions of relapse risk. However, these applications remain investigational. No single mitochondrial parameter currently provides a validated, universal measure of mitochondrial fitness, and differences in sample handling, assay platforms, cellular composition, and biological context may substantially affect results. Prospective studies must therefore establish analytical reproducibility, define clinically meaningful thresholds, and demonstrate whether mitochondrial measurements provide information beyond established clinical, molecular, and immune biomarkers [96].

Several limitations of the current evidence should be emphasized. Much of the mechanistic literature derives from cell lines and experimental models, whereas direct functional measurements of mitochondrial fitness in patients receiving immunotherapy remain limited. Mitochondrial phenotypes can also differ markedly according to tumor lineage, disease stage, treatment history, microenvironmental niche, and immune-cell subset. In addition, mitochondrial respiration, mass, membrane potential, ROS production, and mitochondrial gene expression capture different biological dimensions and should not be treated as interchangeable surrogate measures. The proposed mitochondrial functional immune checkpoint should therefore be considered a hypothesis-generating concept that requires validation across tumor types and therapeutic settings rather than an established clinical classification system.

Taken together, the available evidence supports mitochondrial fitness as an important determinant of both tumor adaptation and immune-cell competence, while highlighting substantial biological heterogeneity and translational uncertainty. Future studies should determine which functional features of mitochondrial fitness best predict immunotherapy response, how these features evolve during treatment, and whether tumor and immune-cell mitochondria can be selectively manipulated without compromising normal tissue or immune function. Such studies will be essential to determine whether the mitochondrial functional immune checkpoint can move from a conceptual model to a clinically actionable component of precision immuno-oncology.

## 8. Conclusions

Mitochondria should no longer be viewed simply as metabolic organelles that supply energy to cancer cells. Their ability to remodel metabolism, maintain redox and structural integrity, regulate apoptosis and stress responses, communicate with neighboring cells, and adapt to therapeutic pressure places them at the intersection of tumor evolution and immune competence. Across cancer types and treatment settings, mitochondrial plasticity can increase tumor resilience, whereas loss of mitochondrial fitness can constrain the ability of T cells and other immune effectors to sustain antitumor activity.

The present Review has intentionally examined these concepts through a predominantly hematological lens. This focus allows mechanistic and translational depth in settings such as acute myeloid leukemia and multiple myeloma, in which mitochondrial dependencies, bone-marrow microenvironmental interactions, residual disease, and immune-based therapies can be considered within a relatively coherent framework. However, the solid-tumor studies discussed throughout the Review—including examples involving mitochondrial transfer, mitochondrial-directed pharmacological strategies, and metabolic engineering of antitumor T cells—indicate that the core principles of mitochondrial adaptation and immune-cell fitness extend beyond hematological malignancies. These examples should be regarded as evidence of broader biological relevance rather than as a comprehensive review of mitochondrial biology in solid tumors.

The mitochondrial functional immune checkpoint proposed in this review is therefore a conceptual framework rather than a new receptor–ligand checkpoint. It describes the integrated requirement for mitochondrial resilience on both sides of the tumor–immune interaction: tumor cells must be rendered sufficiently metabolically vulnerable, while immune effector cells must retain or acquire the mitochondrial competence required for durable function. This perspective may help explain why antigen recognition, immune-cell infiltration, or pharmacological reinvigoration alone does not invariably result in sustained tumor control.

The therapeutic implications are potentially important but remain investigational. Future strategies should aim not for indiscriminate mitochondrial inhibition, but for selective modulation of mitochondrial fitness—weakening tumor-cell adaptability while preserving or enhancing immune-cell metabolic competence. Functional mitochondrial profiling may ultimately complement genomic, immunological, and measurable-residual-disease biomarkers, but standardized assays and prospective clinical validation are required before such approaches can guide treatment selection.

An important next step will therefore be to distinguish conserved mitochondrial mechanisms shared across cancer types from lineage-, microenvironment-, and treatment-specific dependencies. Comparative validation in both hematological and solid malignancies will be necessary to establish the extent to which the mitochondrial functional immune-checkpoint framework can serve as a general principle of precision immuno-oncology.

Ultimately, the clinical value of the mitochondrial functional immune checkpoint will depend on whether this framework can identify actionable differences between tumor-cell and immune-cell mitochondrial states and translate those differences into safer, more durable immunotherapy.

## Figures and Tables

**Table 1:** Mitochondrial-targeting and immune-modulatory strategies: mechanisms, evidence, and translational considerations.

Strategy	Mitochondrial Process/Target	Disease/Model	Therapeutic Context	Main Finding	Evidence Level	Main Limitation/Translational Issue	Reference
IACS-010759	Complex I/OXPHOS	Cancer models with increased oxidative metabolic dependence	Direct OXPHOS inhibition	Reduced mitochondrial respiration and exploited OXPHOS dependence, demonstrating that respiratory metabolism can represent a therapeutic vulnerability	Preclinical + early clinical development	Narrow therapeutic window and potential toxicity of systemic OXPHOS inhibition; metabolic compensation may limit efficacy	[78]
Gboxin	F0 F1 ATP synthase/OXPHOS	Primary mouse and human glioblastoma cells; allograft and patient-derived xenograft models	Direct mitochondrial respiratory inhibition	Selectively impaired oxygen consumption and inhibited glioblastoma growth; a metabolically stable analogue reduced tumor growth in vivo	Preclinical	No established clinical anticancer efficacy; selectivity and systemic tolerability remain to be established	[79]
Mitochondrial translation inhibition	Mitochondrial protein synthesis	Venetoclax-resistant AML models	Tedizolid or doxycycline ± venetoclax	Mitochondrial translation inhibition caused respiratory dysfunction and activation of the integrated stress response and restored venetoclax sensitivity	Preclinical	Potential toxicity to normal hematopoietic and immune cells; pharmacokinetic and treatment-duration issues; clinical efficacy not established	[80]
Urolithin A	Mitophagy/mitochondrial quality control	CD8^+^ T cells; tumor-bearing preclinical models	Immune-cell metabolic conditioning/mitophagy induction	Expanded T memory stem cells, enhanced mitochondrial fitness, and improved antitumor CD8^+^ T-cell activity	Preclinical for cancer immunotherapy; clinical investigation for mitochondrial health	Anticancer efficacy has not been established clinically; optimal dose, schedule, and tumor-versus-immune-cell effects remain uncertain	[81]
ONC206	Mitochondrial ClpP/mitochondrial proteostasis	Hepatocellular carcinoma and other experimental tumor models	ClpP activation	Induced mitochondrial proteolysis, loss of membrane potential, impaired OXPHOS, increased mitochondrial ROS, and cellular stress	Preclinical	Clinical efficacy remains investigational; therapeutic window and patient selection remain undefined	[82]
Venetoclax	BCL-2/mitochondrial apoptosis	AML	Venetoclax + azacitidine	Improved remission and survival compared with azacitidine alone in previously untreated patients with AML who were ineligible for intensive chemotherapy	Clinical	Resistance may involve metabolic rewiring, altered substrate utilization, and alternative anti-apoptotic dependence	[85]
Venetoclax + azacitidine	BCL-2 dependence/mitochondrial metabolism	AML; patient samples and experimental models	Venetoclax + azacitidine	Disrupted energy metabolism and targeted leukemia stem cells, providing a translational link between mitochondrial metabolism and venetoclax sensitivity	Clinical/translational	Residual leukemia may adapt metabolically and develop alternative survival pathways	[50]
Fatty-acid oxidation targeting	Fatty-acid oxidation/mitochondrial substrate utilization	AML; leukemia stem cells	Metabolic inhibition + venetoclax/azacitidine	Increased fatty-acid oxidation contributed to venetoclax resistance; targeting this metabolic adaptation restored sensitivity in experimental models	Preclinical	Metabolic redundancy and compensatory substrate utilization may limit efficacy	[52]
ADSS2 inhibition	De novo AMP biosynthesis/mitochondrial bioenergetics	AML; experimental models	ADSS2 inhibition + BH3 mimetics	Disrupted mitochondrial bioenergetics and restored sensitivity to BH3 mimetics, including venetoclax	Preclinical	Emerging evidence; clinical applicability and selective therapeutic window remain unestablished	[53]
CAR-T metabolic conditioning	Mitochondrial reserve/oxidative metabolism/metabolic flexibility	CAR-T cells	T-cell selection, expansion and metabolic conditioning	Less differentiated memory-associated T-cell populations exhibit greater mitochondrial reserve and metabolic flexibility, supporting persistence and durable antitumor activity	Preclinical/translational	Optimal metabolic phenotype is context dependent and does not necessarily correspond to maximal OXPHOS	[89]
4-1BB CAR costimulation	Mitochondrial biogenesis/fatty-acid oxidation/respiratory capacity	CAR-T cells	CAR design	4-1BB signaling promotes mitochondrial biogenesis, fatty-acid oxidation and respiratory capacity and favors memory-associated differentiation	Preclinical/translational	Effects depend on CAR architecture and cellular context; not equivalent to direct mitochondrial targeting	[76]
CAR-T metabolic fitness	Mitochondrial/metabolic fitness of therapeutic T cells	CLL; CD19 CAR-T cells	Adoptive cellular therapy	Features of the infused product associated with memory differentiation and metabolic fitness correlated with clinical response	Clinical/translational	Association does not establish that mitochondrial parameters are independently predictive or causal	[75]
Mitochondrial transfer	Intercellular mitochondrial trafficking	AML; bone-marrow stromal cells and leukemic blasts	Tumor–microenvironment interaction	Stromal-to-leukemia mitochondrial transfer enhanced oxidative metabolism, redox buffering and leukemia-cell survival during chemotherapy	Preclinical	Directionality is context dependent; indiscriminate blockade could impair potentially beneficial mitochondrial support of immune cells	[51]
Mitochondrial stress/mtDNA–cGAS-STING	mtDNA release/innate immune sensing	Experimental cancer models	Mitochondrial stress-induced immune activation	Mitochondrial perturbation can activate mtDNA-dependent cGAS-STING signaling and promote antitumor immune responses	Preclinical	Context-dependent; excessive mitochondrial injury in immune cells may compromise immune function	[86]
Mitochondrial dynamics targeting	Mitochondrial fission/fusion	Multiple myeloma	Experimental metabolic targeting	Manipulation of mitochondrial dynamics generated metabolic vulnerabilities and antimyeloma activity in experimental models	Preclinical	Disease-specific evidence; clinical feasibility and effects on normal/immune cells remain incompletely defined	[46]
Mitochondrial metabolism in multiple myeloma	Mitochondrial metabolism, proteostasis and redox regulation	Multiple myeloma	Potential combinations with proteasome inhibitors, immunomodulatory agents, CAR-T cells and bispecific antibodies	Identifies mitochondrial metabolic vulnerabilities as potential therapeutic targets and combination opportunities	Review/translational framework	Most proposed strategies remain preclinical and require biomarker-guided clinical validation	[41]

**Table 2:** Potential clinical applications of mitochondrial profiling in precision oncology and immuno-oncology.

Clinical Application	Biological Rationale	Candidate Mitochondrial Features/Biomarkers	Clinical Utility and Therapeutic Implications	References
Baseline tumor metabolic stratification	Heterogeneous mitochondrial states across tumors Effects on bioenergetics, metabolic adaptation, survival, and treatment response	OXPHOS dependence/respiratory capacity Mitochondrial mass/mtDNA copy number Metabolic gene signatures	Stratify tumors by mitochondrial dependence and metabolic flexibility Guide selection for mitochondria-directed or metabolism-based therapies	[29]
Prediction of therapeutic resistance and disease persistence	Mitochondrial plasticity promotes stress adaptation Survival under chemotherapy, targeted therapy, metabolic stress, and immune pressure	OXPHOS/fatty-acid oxidation Antioxidant capacity/mitochondrial biogenesis/mitophagy Anti-apoptotic BCL-2-family dependence	Identify metabolically adapted, drug-tolerant, or therapy-resistant populations Inform combinations targeting mitochondrial fitness alongside conventional or targeted therapies	[27,52]
Mitochondrial characterization of measurable residual disease	Residual malignant cells can retain persistence- and relapse-associated metabolic adaptations Functional stress tolerance is not captured by tumor burden alone	Mitochondrial mass/respiratory capacity/membrane potential OXPHOS activity/ROS handling Mitochondrial quality-control signatures	Add functional mitochondrial characterization to quantitative MRD assessment Refine relapse-risk stratification	[10,96,97]
Assessment of immune-cell mitochondrial competence	Durable antitumor immunity requires sustained activation, proliferation, persistence, and cytotoxicity These functions depend on metabolic competence under stress	Mitochondrial biogenesis/mass Respiratory and spare respiratory capacity/OXPHOS ROS balance	Assess immune-cell functional fitness and persistence Inform metabolic conditioning and selection of more resilient immune-cell populations	[18,19,66]
Prediction and optimization of CAR-T-cell efficacy	CAR-T-cell persistence and antitumor activity depend on differentiation state Mitochondrial fitness and metabolic flexibility shape functional durability	Mitochondrial mass/respiratory and spare respiratory capacity Mitochondrial biogenesis Memory- and exhaustion-associated metabolic programs	Profile CAR-T products for expansion, persistence, and antitumor activity Guide T-cell selection, metabolic conditioning during manufacturing, and mitochondrial-fitness preservation	[72,75,76]
Optimization of T-cell-redirecting bispecific-antibody therapy	Repeated antigen engagement requires sustained endogenous T-cell competence Metabolic fitness influences repeated tumor-cell redirection	Respiratory and spare respiratory capacity ROS regulation Exhaustion-associated metabolic signatures/metabolic flexibility	Assess endogenous T-cell capacity for repeated tumor-cell redirection Design combinations that preserve immune-cell metabolic competence	[77,78]
Characterization of tumor–immune metabolic interactions	Tumor, stromal, and immune cells compete for nutrients and oxygen Metabolic by-products and mitochondrial signaling modulate immune function	Glucose/lactate metabolism/hypoxia signatures Oxidative-stress/mitochondrial-transfer signatures mtDNA- and cGAS–STING-related pathways	Identify metabolic drivers of immune suppression, immune exclusion, and tumor adaptation Prioritize strategies that reduce tumor metabolic pressure while preserving immune-cell function	[16,21,56,57,58]
Monitoring mitochondrial stress and innate immune remodeling	Mitochondrial damage releases mtDNA and generates ROS Innate immune activation reshapes tumor–immune interactions	Cytosolic mtDNA/cGAS–STING activation Mitochondrial ROS/membrane potential Inflammatory and stress-response signatures	Monitor mitochondrial stress, tumor immunogenicity, and immune remodeling Characterize mitochondrial–innate immune signaling for context-specific therapeutic modulation	[21,59]
Evaluation of intercellular mitochondrial transfer	Mitochondrial exchange occurs among tumor, stromal, and immune cells Transfer can alter metabolic fitness, immune function, and therapeutic resistance	Mitochondrial trafficking/tunneling-nanotube activity Donor/recipient mtDNA signatures Mitochondrial-transfer-associated metabolic profiles	Identify microenvironment-driven metabolic rescue and immune dysfunction Distinguish tumor-supportive mitochondrial transfer from beneficial immune-cell interactions as a therapeutic target	[23,25,69]
Functional precision immuno-oncology	Genomic and immune biomarkers provide largely static information Mitochondrial profiling adds a dynamic readout of adaptive capacity and functional fitness	Integrated mitochondrial multi-omics/respiratory assays Mitochondrial mass/membrane potential/substrate use Oxidative-stress signatures/immune-cell metabolic phenotyping	Integrate mitochondrial, genomic, and immune biomarkers for patient stratification and treatment monitoring Guide biomarker-based strategies that exploit tumor mitochondrial vulnerabilities while preserving immune-cell competence	[94,95]

Abbreviations: CAR-T, chimeric antigen receptor T cell; cGAS–STING, cyclic GMP-AMP synthase–stimulator of interferon genes; MRD, measurable residual disease; mtDNA, mitochondrial DNA; OXPHOS, oxidative phosphorylation; ROS, reactive oxygen species. All clinical applications and therapeutic implications listed here are investigational and require prospective clinical validation.

## Data Availability

No new data were created or analyzed in this study. Data sharing is not applicable to this article.
